# Doc2b Ca^2+^ binding site mutants enhance synaptic release at rest at the expense of sustained synaptic strength

**DOI:** 10.1038/s41598-019-50684-1

**Published:** 2019-10-08

**Authors:** Quentin Bourgeois-Jaarsma, Matthijs Verhage, Alexander J. Groffen

**Affiliations:** 1 Department of Functional Genomics, Faculty of Science, Center for Neurogenomics and Cognitive Research, Vrije Universiteit, De Boelelaan 1085, 1081HV Amsterdam, The Netherlands; 2 Department of Clinical Genetics, Center for Neurogenomics and Cognitive Research, VU Medical Center, De Boelelaan 1085, 1081HV Amsterdam, The Netherlands

**Keywords:** Exocytosis, Molecular neuroscience

## Abstract

Communication between neurons involves presynaptic neurotransmitter release which can be evoked by action potentials or occur spontaneously as a result of stochastic vesicle fusion. The Ca^2+^-binding double C_2_ proteins Doc2a and –b were implicated in spontaneous and asynchronous evoked release, but the mechanism remains unclear. Here, we compared wildtype Doc2b with two Ca^2+^ binding site mutants named DN and 6A, previously classified as gain- and loss-of-function mutants. They carry the substitutions D218,220N or D163,218,220,303,357,359A respectively. We found that both mutants bound phospholipids at low Ca^2+^ concentrations and were membrane-associated in resting neurons, thus mimicking a Ca^2+^-activated state. Their overexpression in hippocampal primary cultured neurons had similar effects on spontaneous and evoked release, inducing high mEPSC frequencies and increased short-term depression. Together, these data suggest that the DN and 6A mutants both act as gain-of-function mutants at resting conditions.

## Introduction

Regulated exocytosis is strictly dependent on *Soluble N-ethylmaleimide-sensitive-factor Attachment protein REceptor* (SNARE) proteins, Ca^2+^-sensors and a number of accessory proteins^1^. Neurotransmitter release is either triggered by action potentials (APs)^1–5^ or occurs spontaneously at resting membrane potential^3,6^. Evoked release consists of synchronous and asynchronous release components^2,7,8^. Fast, synchronous release triggered by local Ca^2+^ influx (nano & micro-domain) occurs in less than a millisecond^3,9^ and is governed by the fast Ca^2+^ sensors Syt-1, 2 or 9^10^. Another class of high affinity Ca^2+^ sensors with slow kinetics such as Syt-7 mediates asynchronous release^8,11–13^ and synaptic plasticity^14^. In synapses lacking the fast sensor, diminished synchronous release is accompanied by increased asynchronous release as shown in Syt-1^15–17^ and Syt-2^18,19^ mutant mice.

Unlike evoked release, spontaneous release is AP-independent and occurs as a stochastic process with a probability that appears to be partially regulated by intracellular Ca^2+^ ^20–22^. Spontaneous release is important for nervous system functioning as it is involved in synapse maturation, maintenance and synaptic plasticity^23–26^. Like asynchronous release, its frequency is suppressed by Syt-1 and Syt2^22,27,28^ and stimulated by double C_2_ (Doc2) proteins^21,27,29^.

Doc2a, -b and –c isoforms together constitute the Doc2 protein family. Doc2a is mainly expressed in the adult brain while Doc2b is more widely expressed in the nervous system and various neuroendocrine tissues^30,31^. Both Doc2a and –b contribute to spontaneous release as shown in knockout and knock-down models^21,29^. A recent study suggested that glutamatergic and GABAergic events are driven by the expression of Doc2a and –b respectively, although both isoforms are functionally redundant and can rescue both miniature excitatory and inhibitory post-synaptic current (respectively mEPSC and mIPSC) frequencies^27^.

**Figure 1:**
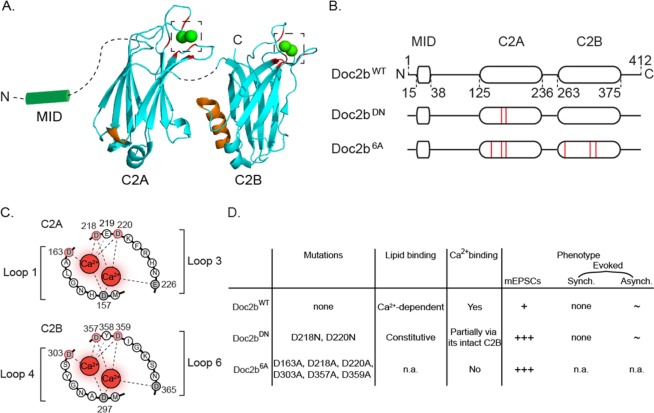
Molecular and phenotypic properties of Doc2b and its Ca^2+^-binding site mutants. (**A**) Cartoon showing C_2_ domain structures of Doc2b based on crystallography^51^. Aspartates involved in Ca^2+^ binding are marked in red; poly-lysine sequences for SNARE complex and PIP_2_ interaction are marked in orange^66^. Note that the poly-lysine region is oriented opposite to the Ca^2+^-binding aspartates. Dashed lines represent linker sequences between domains. Dashed squares highlight Ca^2+^-binding pockets enlarged in C. (**B**) Linear representation of Doc2b^WT^ and two previously investigated mutants Doc2b^DN^ and Doc2b^6A^ (red lines indicate amino acid substitutions). (**C**) Aspartates substituted in Doc2b^DN^ (D218, 220N) or Doc2b^6A^ (D163, 218, 220, 303, 357, 359A). (**D**) Summary of functional effects of Doc2b^DN^ and Doc2b^6A^ mutations. Ca^2+^-binding capacity was assessed by tryptophan fluorescence measurements^49^ for Doc2b^6A^ and isothermal titration calorimetry (ITC) measurement for Doc2b^DN^ (termed CLM mutant)^44^. Synaptic release phenotypes were determined by electrophysiology in cultured neurons. Doc2b^WT^ supports spontaneous release (marked as ‘+’ in the table). Its role in asynchronous release is observed in some but not all systems^29,44,47^ (marked as ‘~’). It does not function in synchronous release (‘none’). Doc2b^DN^ constitutively binds phosphatidylserine-containing membranes^44,48^ and partially binds Ca^2+^ via its intact C_2_B domain^44^. It increases spontaneous release frequency (+++)^21,29^ and is implicated in asynchronous release in some but not all studies (~)^21,44^. No effect was noticed on synchronous release (none). Doc2b^6A^ was shown have an abrogated Ca^2+^-binding^29^ capacity but its lipid association activity was not reported (n.a.). This mutant increased spontaneous release frequency^29^ (+++) but its effect on evoked synchronous and asynchronous release were not investigated (n.a.). See^21,29,44^ for more details.

In cell-free assays, Doc2b interacts with the SNARE complex via a polybasic sequence (Fig. 1A, orange) and promotes fusion of SNARE-liposomes^21,32^. The polybasic sequence also enables Doc2b to bind PI(4,5)P_2_, a phospholipid enriched on the cytoplasmic leaflet of the plasma membrane^33^. On the opposite site of the C_2_ domain structures, negatively charged residues (Fig. 1A, red) bind to phosphatidylserine-containing membranes in a Ca^2+^-dependent manner^34^. As shown by selective mutations of the polybasic motif versus the Ca^2+^-binding loops, SNARE complex and phosphatidylserine binding can happen in parallel, independently^21^. Indeed, the inhibition of SNARE interaction in the K237,319E mutant does not affect liposome binding. Conversely a mutant with a loss of hydrophobic residues at the Ca^2+^ binding site shows no deficiency in SNARE interaction^21^.

The N-terminal domain of Doc2a/b interacts with Munc13 via a Munc13 interacting domain (MID; Fig. 1A,B) in HEK293 cells^35^, PC12 cells^36–38^ and neurons^39,40^. This interaction is sufficient for co-translocation of Munc13 together with Doc2 upon stimulation with phorbol ester (a diacylglycerol homologue)^35,39,41^. Phorbol esters potentiate exocytosis in a Ca^2+^-independent way relying on the Doc2/Munc-13 interaction^39,41,42^. Consistently, Doc2 overexpression causes a Ca^2+^-independent, Munc-13 dependent release increase upon phorbol ester stimulation^38^ and conversely, blockade of the Doc2-Munc13 interaction by synthetic peptides abolishes phorbol ester potentiation^39^. Munc13-1 is necessary for the Doc2b-induced priming of secretory granules in chromaffin cells^43^. However, alteration of this interaction by mutations in the MID domain has no effect on Ca^2+^-induced Doc2b migration to the membrane^41,44^. Hence, Doc2b could support exocytosis by both of two mechanisms: (i) together with Munc-13 for vesicle priming or superpriming; (ii) Ca^2+^-dependently by enhancing membrane fusion.

Doc2a/b have a high Ca^2+^ affinity with half-maximal membrane binding at 450 nM and 175 nM respectively in chromaffin cells^45^. Ca^2+^ binding onto their C_2_ domains requires five conserved acidic residues (Fig. 1C) in close proximity to hydrophobic loops which interact with the membrane after Ca^2+^ activation, resulting in reversible translocation^41,45^. A similar mechanism occurs in Syt-1^46^. In fact, Doc2b and Syt-1 compete for SNARE protein binding^21,32^, which suggests a partially shared mechanism in Ca^2+^-secretion coupling as recently mentioned for spontaneous release^27^.

It is still debated whether Doc2b acts as a direct Ca^2+^-sensor or as a structural element supporting Ca^2+^-dependent secretion by another process. Neutralization of two critical aspartates D218 and D220 in the C_2_A domain of Doc2b (Fig. 1B–D) induces Ca^2+^-independent membrane-binding of the domain^47,48^. This mutant, designated Doc2b^DN^, was therefore considered a gain-of-function mutant. When Doc2b^DN^ expression caused a rise of the spontaneous release rate (mEPSCs), this was taken to support a role as Ca^2+^ sensor^21,45^. Another Ca^2+^-ligand mutant designated Doc2b^6A^, in which six aspartates were substituted by alanines (Fig. 1B–D), is unable to bind Ca^2+^ and therefore considered a loss-of-function mutant^29^. This mutant still rescues spontaneous release in Doc2 double knock-down neurons (Fig. 1D), suggesting a Ca^2+^-independent mechanism. Nevertheless, a recent study suggested that this mutant may enhance spontaneous release by mimicking a Ca^2+^-bound state^27^.

As another point of debate, several studies reported that Doc2a and -b both contribute to asynchronous release in hippocampal network cultures^32,44,47^. The asynchronous component was further enhanced by expression of Ca^2+^-binding mutants of Doc2b. Other studies in autapse cultures of hippocampal neurons reported no effect of Doc2b on asynchronous release^21,49^ leaving room for debate whether Doc2b acts selectively on spontaneous release, selectively on asynchronous release, or takes part in both processes. A schematic overview of the various observed functions on the molecular and cellular level is depicted in Fig. 1D.

To clarify the inconsistencies regarding the gain- or loss- of function classification of Doc2b mutants, as well as their role as Ca^2+^ sensors in spontaneous and asynchronous release, we directly compared both mutants with wildtype Doc2b. Their Ca^2+^-dependent membrane-binding activity, subcellular localization and their effects on spontaneous and evoked neurotransmission demonstrated that both mutants showed a gain-of-function behavior at resting Ca^2+^ concentrations. These results reconcile previous findings on spontaneous and evoked release and support the idea that Doc2b can affect both spontaneous release and short-term plasticity.

## Results

### Aspartate substitutions in Doc2b^DN^ and Doc2b^6A^ cause constitutive membrane association in resting neurons

**Figure 2:**
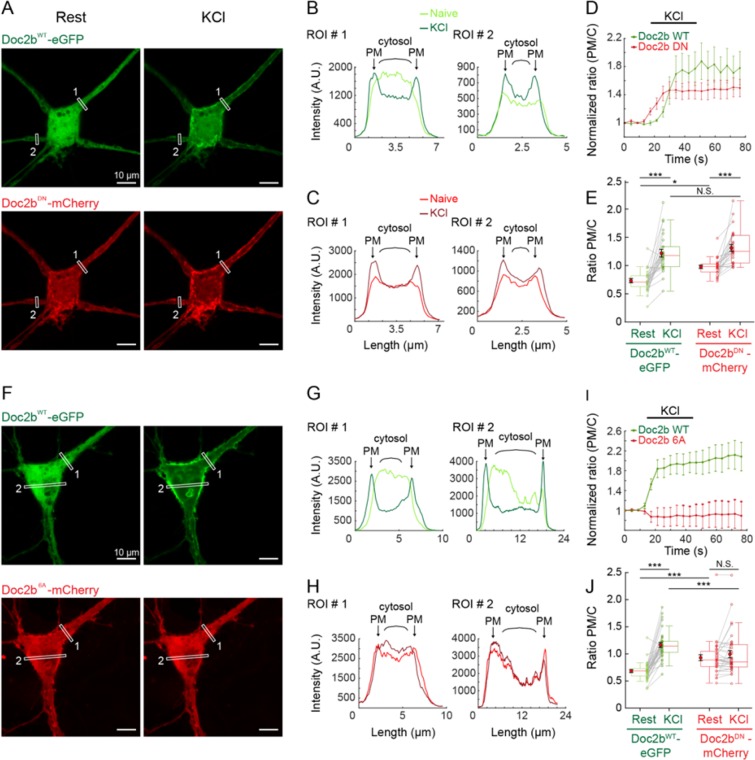
Ca^2+^-binding site mutants Doc2b^DN^ and Doc2b^6A^ show increased plasma membrane binding at rest but different activity during stimulation. (**A**) Live confocal microscopy of hippocampal neurons cultured in low density networks co-expressing Doc2b^WT^ tagged with eGFP (green) and either Doc2b^DN^ or Doc2b^6A^ fused to mCherry (red). Both channels were simultaneously imaged for 15 s at rest and then stimulated with 60 mM KCl in the extracellular medium. Representative images were obtained by averaging 4 sequential images each for the resting (naïve) and stimulated state (KCl). (**B**,**C**) Representative line scans from 2 of 5 ROIs per cell with a thickness of 10 pixels placed on neurites (see white rectangles in **A**) showing the fluorescence intensity distribution at rest (light-colored profiles) and after stimulation (dark-colored) for Doc2b^WT^-eGFP (**B**) and Doc2b^DN^-mCherry (**C**). Arrows indicate the plasma membrane (PM); curly brackets indicate cytosol (**C**). (**D**) Average PM/C intensity ratio from 5 ROIs of the representative neuron, normalized to the starting value. Both Doc2b^WT^–eGFP and Doc2b^DN^-mCherry translocated upon KCl stimulation. (**E**) The PM/C fluorescence ratio was averaged from 6 neurons (N = 6) each containing 2 to 6 ROIs (n = 26 ROIs in total). (**F-J**) The same methodology was applied to neurons co-expressing Doc2b^WT^-eGFP and Doc2b^6A^-mCherry. (**F**) Representative example showing WT and 6A behavior at rest and upon stimulation. (**G,H**) Line scan profiles for Doc2b^WT^-eGFP (**G**) and Doc2b^6A^-mCherry (**H**). (**I**) Normalized PM/C intensity ratio averaged from 5 ROIs in the representative neuron. (**J**) PM/C ratio quantification from n = 36 ROIs from N = 11 neurons, showing a strong translocation of Doc2b^WT^-eGFP while in the same cell, Doc2b^6A^-mCherry appears insensitive to stimulation. For (**D**–**I**) and (**E**–**J**) data are represented as mean ± SEM. Friedman ANOVA, paired repeated Post-hoc tests (see Supplementary Table 1).

Activity-dependent plasma membrane binding is an established feature of Doc2b^45^. We first tested the Ca^2+^-dependent membrane binding of the Doc2b^DN^ and Doc2b^6A^ mutants, previously described to be gain- and loss-of function variants respectively^21,29^ under resting conditions and during stimulation (Fig. 2A–F). To assess the activity dependence of membrane association for both Doc2b^WT^ and mutants simultaneously, we performed confocal live imaging at rest and during stimulation with a 60 mM KCl puff for 30 s in WT neurons (Fig. 2). To internally control cell-to-cell variation, mCherry-tagged mutants (Doc2b^DN^-mCherry or Doc2b^6A^-mCherry; see Fig. 2A,F) were co-expressed with eGFP-tagged Doc2b^WT^ in the same neurons. At resting conditions, eGFP-tagged Doc2b^WT^ was mostly localized throughout the cytosol (Fig. 2A,F, top panels) but mCherry-tagged Doc2b^DN^ (Fig. 2A,F bottom panel) and Doc2b^6A^ (Fig. 2F bottom panel) both showed plasma membrane (PM) enrichment. The WT and mutant phenotypes were clearly discernable in line scan profiles of ROIs placed on neurites or the soma (Fig. 2B,C,G,H respectively, left panel), consistent with previous studies^43,45^. During prolonged chemical depolarization by KCl, Doc2b^WT^ showed a clear plasma membrane association visible as two peaks of fluorescence in the line scans (Fig. 2B,G, arrows). Quantification of the ratio of PM and cytosolic (C) signal from 5 ROIs showed a gradual translocation of Doc2b^WT^ (Fig. 2D). The distribution of Doc2b^DN^ (Fig. 2C) and its normalized PM/C ratio (Fig. 2D) confirmed its partial constitutive membrane bound state, while the increase upon KCl application indicated remaining sensitivity of this mutant to Ca^2+^. Quantification of the PM/C ratio from 26 ROIs (Fig. 2E) on timelapse movies from 6 neurons enables to conclude that Doc2b^DN^ is significantly more PM-enriched than Doc2b^WT^ at rest (Fig. 2E “rest”, 0.73 ± 0.04 vs 0.98 ± 0.03 for WT and DN respectively, see Supplemental Table 1). Yet, Doc2b^DN^ still senses neuronal activity as it significantly translocates (Fig. 2E) upon KCl stimulation.

Also the Doc2b^6A^ mutant showed constitutive PM association, quantitated in 36 ROIs from 11 neurons (Fig. 2J, “rest”, 0.68 ± 0.03 for WT and 0.93 ± 0.05 for 6A). In contrast to Doc2b^DN^, PM enrichment of Doc2b^6A^ was not significantly increased after KCl application (Fig. 2J), suggestion a loss of Ca^2+^ sensitivity.

Thus, in living neurons, both mutants exhibit increased plasma membrane binding at rest while the DN but not the 6A mutant still shows some activity-dependency. The different translocation behavior of Doc2b^WT^ and mutants co-expressed in the same neurons provides an internal control for cytosolic Ca^2+^ elevation and precludes potential effects of di- or multimerization.

### Phospholipid-binding properties of Doc2b mutants

**Figure 3:**
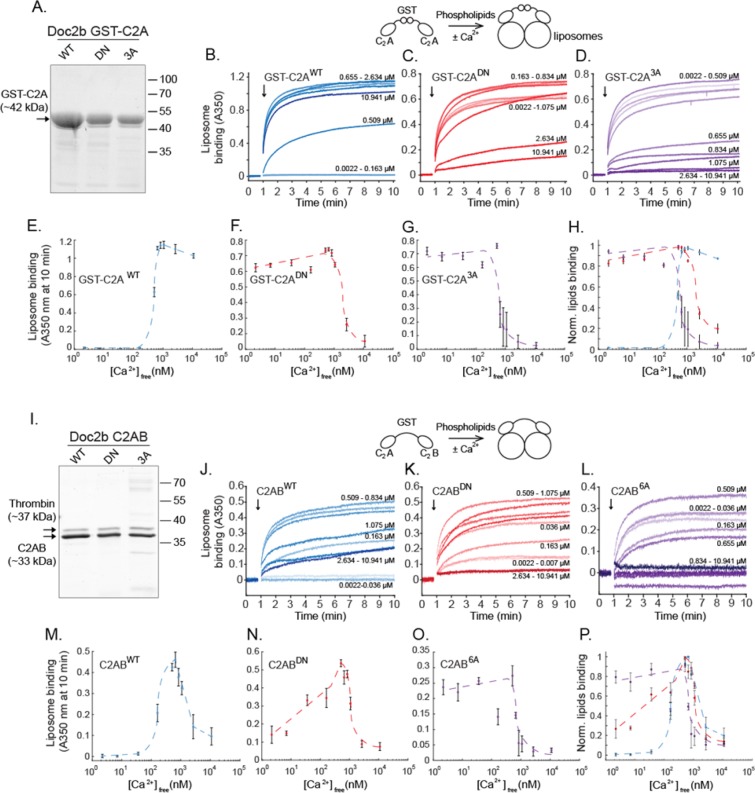
Ca^2+^ binding site mutations cause constitutive phospholipid binding of C_2_A (**A**–**H**) and C_2_AB fragments (**I**–**P**) of Doc2b. (**A**) Recombinant GST-C_2_A fusion protein of wildtype and mutants Doc2b visualized in SDS-PAGE by Sypro Ruby staining. (**B**–**D**) The addition of GST-C_2_A fragments (arrows) to 75% DOPC/25% DOPS-containing liposomes causes liposome aggregation. Kinetic measurements of absorbance at λ = 350 nm were performed in various free Ca^2+^ concentrations in calibrated Ca^2+^/EGTA solutions. (**E**–**H**) Ca^2+^-dependence of phospholipid binding by GST-C_2_A^WT^, GST-C_2_A^DN^ and GST-C_2_A^3A^. Note that C_2_A^3A^ corresponds to the Doc2b^6A^ mutant as it contains only 3 substitutions in the C_2_A domain. (**I**) Recombinant C_2_AB fragments of Doc2b^WT^, Doc2b^DN^, and Doc2b^6A^. (**J**–**L**) Kinetic absorbance measurements of 75% DOPC/25% DOPS liposomes. (**M**–**P**) Ca^2+^-dependence of liposome aggregation activity for each C_2_AB construct. At low (<50 nM) [Ca^2+^]_free_, both mutants showed increased phospholipid binding activity while the wildtype constructs did not. However, in high [Ca^2+^]^free^ conditions (>1 µM), a strong reduction in activity was observed for all constructs except C_2_A^WT^. These two different phases could correspond to a resting and activated state of the protein according to [Ca^2+^]_i_ in the active zone of living neurons and [Ca^2+^]_free_ in our *in-vitro* study. Data are represented as mean ± SEM from N = 5 and N = 4 independent measurements for GST-C_2_A and C_2_AB recombinant fragments respectively. Dashed lines indicate manually drawn trendlines used as visual aids.

The altered subcellular localization of Doc2b^DN^ and Doc2b^6A^ could result from altered C_2_-phospholipid interactions. To investigate this, wildtype and mutant C_2_A and C_2_AB fragments were expressed as recombinant proteins in bacteria. The mutants were expressed to the expected molecular mass as verified by SDS-PAGE (Fig. 3A,I). C_2_-phospholipid binding was measured in a liposome aggregation assay^50^, using calibrated EGTA-buffered Ca^2+^ solutions and liposomes composed of 25% DOPS and 75% DOPC^48^. To test phospholipid binding by the C_2_A domain, the N-terminal glutathione-S-transferase (GST) tag used for the protein purification was preserved to enable C_2_A self-association. Phospholipid binding causes liposome clustering which can be measured as an absorbance increase at 350 nm. Addition of a GST-C_2_A^WT^ protein fragment to a Ca^2+^-containing solution caused rapid liposome clustering (Fig. 3B,E). As a control, GST alone did not cause liposome aggregation (Supplemental Fig. S1). This activity was strictly Ca^2+^- and protein-dependent and followed a sigmoid dose dependence with an EC_50_ of 435 ± 31 nM in line with previous reports^48^. GST-C_2_A^WT^ remained membrane-bound at high [Ca^2+^]_free_ (1–10 µM). In contrast, both GST-C_2_A^DN^ and GST-C_2_A^3A^ showed a similar strong phospholipid binding at low [Ca^2+^]_free_ (0–500 nM; Fig. 3C–D,G,H). At higher [Ca^2+^]_free_, both mutants displayed a large decrease in phospholipid association (Fig. 3C,D,G,H).

To test phospholipid aggregation with full length C_2_AB fragments, the GST tag was cleaved off by thrombin (Fig. 3I; see Methods for details). Thrombin has a specific recognition sequence that is unique in the recombinant protein which shows the expected migration pattern in SDS-PAGE. Liposome clustering by C_2_AB^WT^ increased Ca^2+^-dependently to reach a half-maximum at 176 ± 38 nM (Fig. 3J–M) consistent with reported data^44,45,51^. Maximum activity occurred at approximately 700 nM, followed at higher [Ca^2+^]_free_ by a strong drop in the absorbance signal. At the lowest tested [Ca^2+^]_free_ of 2.2 nM, the C_2_AB^DN^ fragment already showed partial binding activity (Fig. 3K–N). This activity appeared to be partially Ca^2+^-dependent and presumably reflects Ca^2+^-dependent activity from the intact C_2_B domain. At high Ca^2+^ concentrations above 500 nM, a decrease was observed reminiscent of the data obtained with C_2_AB^WT^. The C_2_AB^6A^ fragment showed near-complete liposome binding at the lowest [Ca^2+^]_free_ and no prominent Ca^2+^ dependency in the 0–500 nM range of [Ca^2+^]_free_ (Fig. 3O), followed again by a signal decrease above 500 nM. We did not test liposome aggregation activity induced by the GST-C_2_B domain because Doc2b^DN^ does not contain any mutation in its C_2_B domain.

Taken together, in this cell-free liposome clustering assay, both Doc2b^DN^ and Doc2b^6A^ behave as a gain-of-function mutant at low [Ca^2+^]. However, mutant C_2_A fragments display a loss of phospholipid association at high [Ca^2+^]. This loss was present for all WT and mutant full length C_2_AB constructs. C_2_AB^DN^ and ^6A^ mutations seem to increase the apparent membrane affinity of the protein in low [Ca^2+^] conditions but not in high [Ca^2+^] conditions.

### Doc2b^DN^ or Doc2b^6A^ mutants have no effect on synaptogenesis

**Figure 4:**
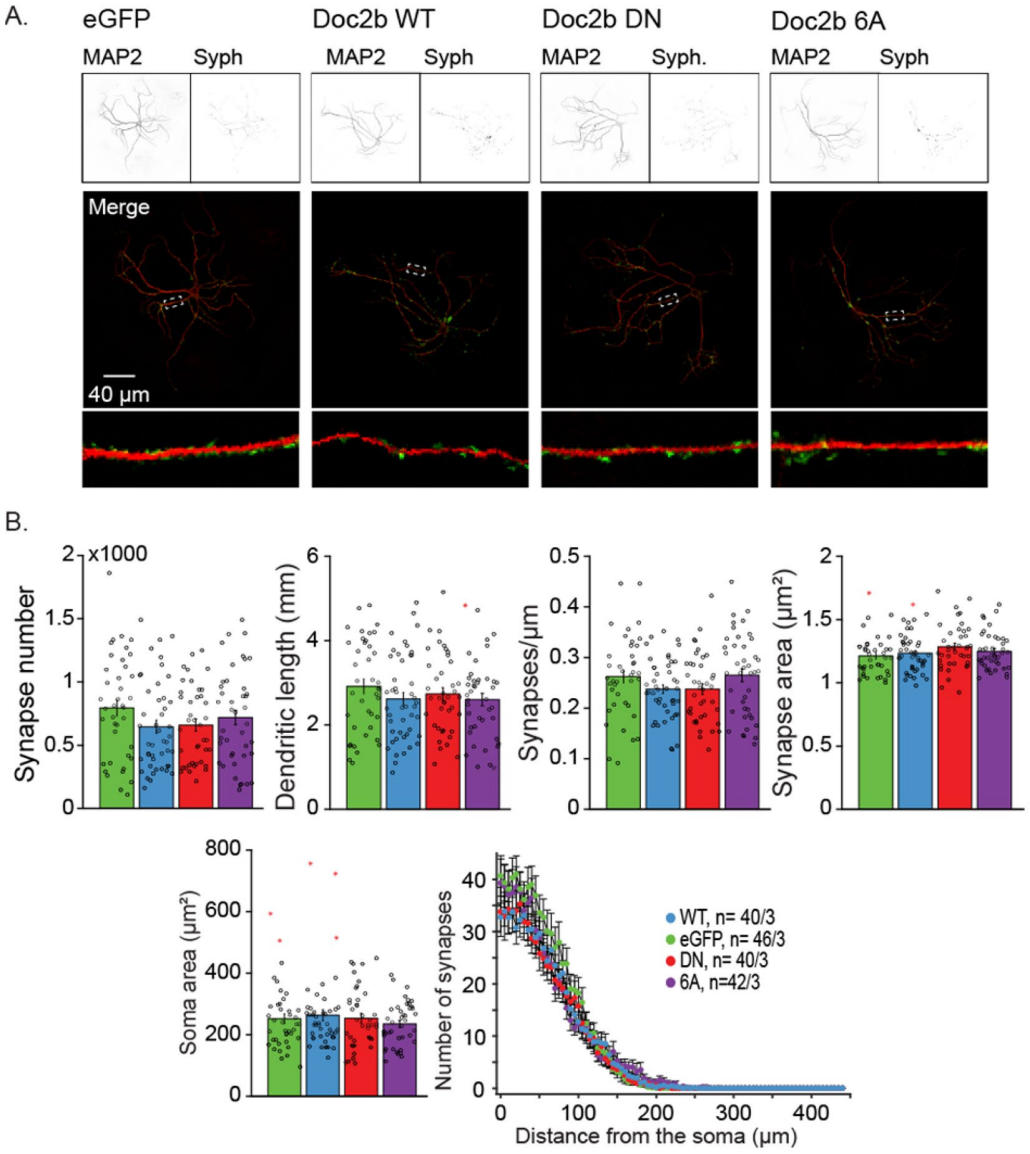
Overexpression of Doc2b^WT^, Doc2b^DN^, and Doc2b^6A^ does not affect the morphology or synapse number in wildtype neurons. (**A**) Confocal images from autaptic wildtype neurons expressing Doc2b constructs or control (eGFP). MAP2 (top left) immunostaining reveals dendritic morphology of the neurons. Synaptophysin (Syph; top right) stains for presynaptic active zones. White rectangles on merged images depict zoomed in dendritic areas (bottom). (**B**) Quantification of synapses and morphologic characteristics as previously described^65^. Data are represented as mean ± SEM from the indicated number of cells over 3 independent experiments. Kruskall Wallis ANOVA and one way repeated ANOVA, Tukey’s Post-hoc tests. Red stars signs indicate outliers. The number of cells (n) and the number of independent experiments (N) are indicated as “n/N”.

Doc2b is temporally and spatially regulated during the embryonic and early postnatal phase^31^ suggesting a role in neuronal development and synaptogenesis. In addition, Syt-7, another high affinity Ca^2+^-sensor which shares homology with Doc2b, has been implicated in neurite outgrowth^52^. One plausible explanation for the high frequency of spontaneous release in Doc2b^DN^ and Doc2b^6A^ expressing cells would be that those mutations affect neurogenesis or development and increase the synaptic density of neurons via altered membrane trafficking. To evaluate this possibility, we performed immunostainings for the synaptic vesicle marker Synaptophysin and the dendritic neuronal marker microtubule associated protein 2 (MAP-2, Fig. 4A). Quantitative morphometry of autaptic neurons expressing Doc2b^WT^ and mutants did not reveal significant changes in synaptic density, dendritic length, dendritic synapse density, synapse area, soma area or synapse distance from the soma (Fig. 4B). Thus, the spontaneous release rise induced by Doc2b mutants is not caused by developmental dysregulation.

### Doc2b^DN^ or Doc2b^6A^ mutants enhance spontaneous release in Doc2-deficient neurons

**Figure 5:**
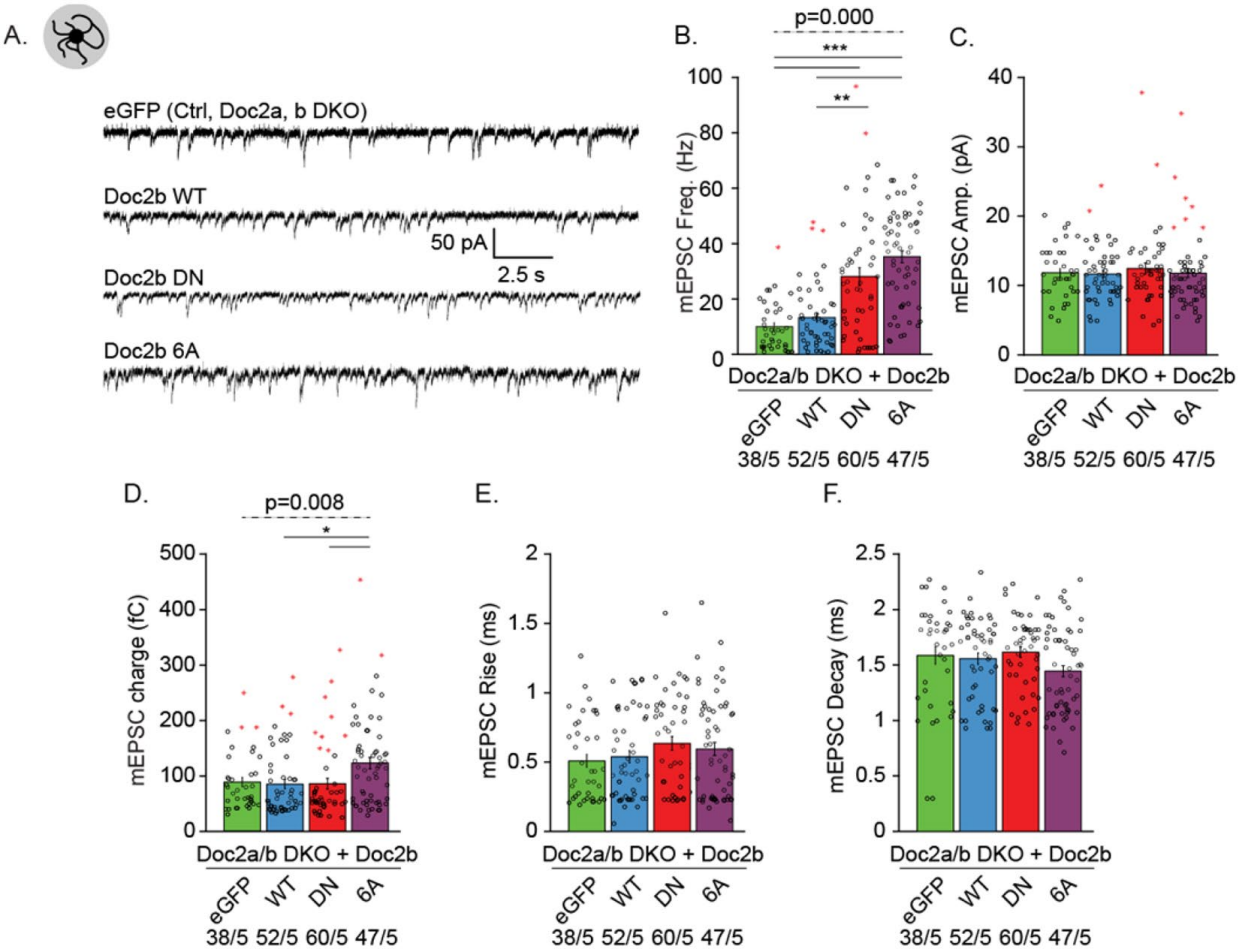
Doc2b Ca^2+^-binding-site mutants enhance the frequency of spontaneous release in Doc2-deficient hippocampal neurons. (**A**) Representative mEPSC recordings in autaptic neurons from Doc2a & b double knockout mice (DKO) overexpressing eGFP (ctrl), Doc2b^WT^, Doc2b^DN^ or Doc2b^6A^. (**B**–**F**) Quantification of spontaneous neurotransmitter release frequency (**B**) and postsynaptic features such as amplitude (**C**), charge (**D**), rise (**E**) and decay time (**F**). Data are represented as mean ± SEM; Kruskall Wallis ANOVA, Pairwise Post-hoc tests (*p<0.05; **p<0.01 ***p<0.005). Red stars signs indicate outliers (included in the analysis). For each group, the number of recordings (n) and the number of independent experiments (N) are indicated.

To measure the effect of Doc2b on synaptic activity and rule out possible effects of endogenous Doc2b in our experiments, we investigated spontaneous release in Doc2a/b double knock-out (DKO) neurons cultured on glial micro-islands (Fig. 5). DKO neurons were rescued with Doc2b^WT^, Doc2b^DN^ or Doc2b^6A^ and expression levels were confirmed by immunoblotting (Fig. S2A). Western blot confirmed the low expression level of endogenous Doc2b^31^ which was detectable in lysate from brain and cultured cortical neurons (Fig. S2C). Viral transduction induced higher Doc2b levels compared to endogenous levels (Fig. S2).

Expression of Doc2b^WT^ increased the average mini frequency from 10 ± 1.4 to 13 ± 1.6 Hz in DKO cells (Fig. 5A,B). This trend is similar to previous observations^21^. Both mutants caused a strong increase in the mEPSC frequency (Fig. 5A,B) while the amplitude, rise and decay (Fig. 5C–F) were unaffected. Quantification of the mEPSC charge showed a small but significant increase for Doc2b^6A^ expressing neurons compared to other groups (Fig. 5D, effect size: 0.838, Supplementary Table 1, page 1, D). The effect size was low and may be attributable to less accurate fitting of exponential decay curves in groups with extremely high mEPSC frequencies. This interpretation is consolidated by our investigation in network and autaptic cultures of WT hippocampal neurons where no effect was observed on mEPSC charge. Western blots from hippocampal WT neurons confirmed the viral overexpression (Fig. S2B). In both WT networks and autapses, overexpression of Doc2b^DN^ or Doc2b^6A^ induced an approximately 3-fold increase of the mEPSC frequency (Fig. S3; see Supplementary Table 1 for statistical tests), similar to the results in DKO neurons. In contrast to overexpression in DKO neurons, Doc2b^WT^ expression in WT neurons did not increase the frequency of miniature excitatory postsynaptic currents (mEPSCs), consistent with previous observations^21^. Note that in neuronal networks, spontaneous release was measured in presence of 1 µM tetrodotoxin (TTX) to block voltage-gated sodium channels.

Taken together, the aspartate substitutions in Doc2b^DN^ and Doc2b^6A^ have similar gain of function effects on the spontaneous release frequency, which confirms previous observations^21,27,29^. The effect of mutant Doc2b on spontaneous mEPSC frequency parallels the alteration at rest in *in vitro* plasma membrane binding and phospholipid clustering (i.e. a gain of function in resting conditions). We conclude that Doc2b^DN^ and Doc2b^6A^ both act as dominant positive mutants that increase the probability of quantal release at rest as those mutants have no effect on synaptic density (Fig. 4).

### Doc2b^DN^ or Doc2b^6A^ overexpression alters evoked release

**Figure 6:**
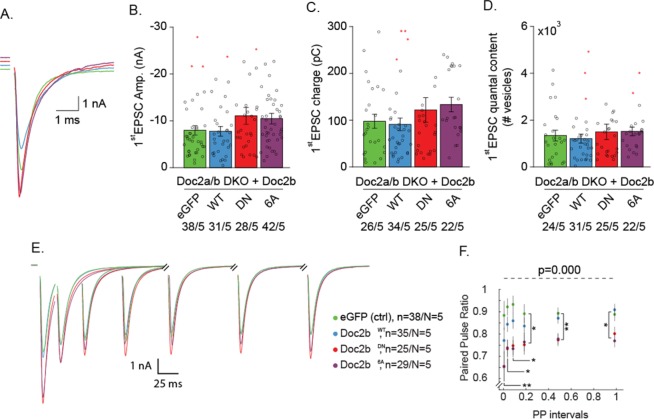
Ca^2+^ binding site mutants Doc2b^DN^ and Doc2b^6A^ enhance short-term depression in Doc2-deficient neurons. (**A**) Representative example traces of single EPSC from DKO neurons overexpressing Doc2b^WT^ or mutants. (**B**) Quantification of 1^st^ EPSC amplitude and (**C**) charge. (**D**) Quantal release or number of vesicles released during the 1^st^ EPSC calculated as EPSC charge divided by mEPSC charge. (**E**) Averaged recordings from paired-pulse stimulations and (**F**) ensuing paired-pulse ratio. Data are represented as mean ± SEM; Kruskall Wallis ANOVA and one way repeated ANOVA, Pairwise Post-hoc tests (*p < 0.05, **p < 0.01, ***p < 0.005). Red stars indicate outliers which were not excluded in the analysis. For each group, the number of recordings (n) and the number of independent experiments (N) are indicated as “n/N”.

The above effects on spontaneous release prompt the question whether the Doc2b mutants also affect synaptic release and plasticity during neuronal activity. We next recorded evoked neurotransmitter release or excitatory post-synaptic currents (EPSCs) induced by a single action potential (AP) or paired APs at varying intervals in DKO autaptic neurons. Expression of Doc2b^WT^ or mutants in DKO cells did not significantly affect the 1^st^ evoked EPSC charge or amplitude (Fig. 6A–C). We then proceeded with paired stimuli given at various intervals of 20–1000 ms (Fig. 6E). The paired pulse ratio, which is the ratio between the EPSC amplitude of the 2^nd^ relative to that of the 1^st^ stimulus, showed stronger depression induced by overexpression of either Doc2b mutant (Fig. 6E,F), most notably in the 20–200 ms interval range (Fig. 6E). The single EPSC quantal content was calculated from the ratio of miniature on evoked EPSC charge to estimate the number of vesicles released during a single AP. The quantal content of single EPSCs was not significantly changed (Fig. 6D).

This phenotype was partially corroborated by a similar investigation in WT autaptic neurons (Fig. S4A–D), wherein the paired-pulse ratio (PPR) again showed stronger synaptic depression for both mutants. Higher single EPSC amplitudes combined with a reduced PPR are usually a characteristic of increased release probability (P_vr_), although alternative explanations exist. Unlike in DKO neurons, WT cells expressing Doc2b^DN^ or Doc2b^6A^ mutant showed a higher 1^st^ evoked EPSC charge (Fig. S4). The 1^st^ EPSC quantal content was significantly larger in WT cells expressing Doc2b^6A^ (Fig. S4E). Whereas both DKO and WT neurons displayed more depression during the paired pulse stimulation, the immediate release during a single AP was only affected by Doc2b^6A^ in WT neurons. Possibly, cumulative effects of Doc2b^6A^ and endogenous Doc2b may contribute to this effect. Besides this difference, our results from DKO and WT cells show strikingly similar effects of both mutants on short-term plasticity during paired pulse stimulation.

Overexpression of Doc2b^WT^ in both DKO and WT neurons uncovered a slight reduction in first evoked amplitude and charge compared to control cells expressing eGFP. In chromaffin cells Doc2b^WT^ was shown to disperse syntaxin-1 from plasma membrane clusters, thereby inhibiting Ca^2+^ currents through voltage gated Ca^2+^ channels (VGCCs)^53^. To test if a similar mechanism occurs in neurons we compared the EPSC amplitudes induced by a single AP and a subsequent treatment with calcimycin in wildtype autaptic neurons expressing either eGFP control or overexpressing Doc2b^WT^. Calcimycin is a Ca^2+^ ionophore which bypasses VGGCs, and triggers exocytosis by an artificial Ca^2+^ influx. No significant change was observed in the EPSC amplitude, nor in the charge transfer induced by calcimycin (Fig. S5A–D), indicating that Doc2b does not inhibit synaptic strength by modulating Ca^2+^ influx in neurons.

### DKO neurons expressing mutant Doc2b^DN^ and ^6A^ suffer from faster synaptic depression during repetitive stimuli

**Figure 7:**
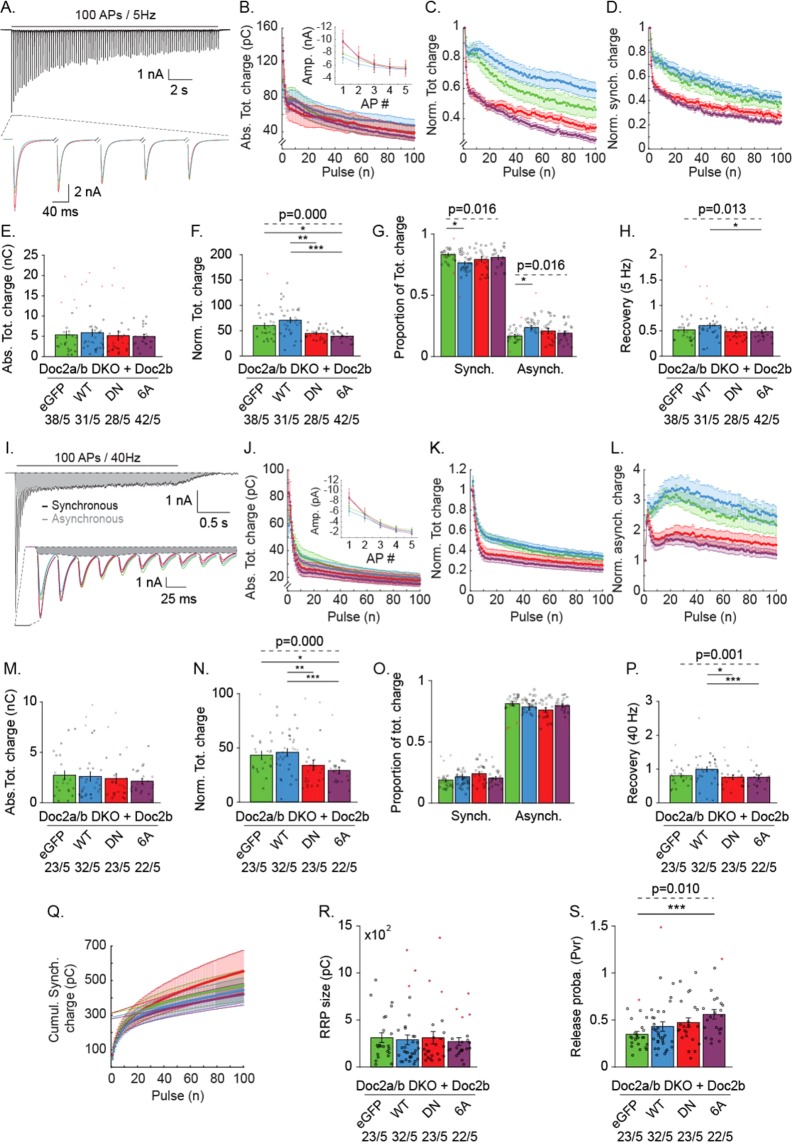
Doc2b mutants affect rundown of EPSC charge during repetitive stimulation in DKO neurons. (**A**) Averaged trace from stimulation of 100 APs at 5 Hz of control DKO neurons (top) and zoom-in on the first five EPSCs for all groups (bottom). (**B**) Absolute total charge rundown together with absolute amplitude for the first five AP (inset). (**C**) Normalized total charge, (**D**) Normalized synchronous charge, (**E**) Absolute total charge and (**F**) Normalized total charge from the entire 5 Hz train. (**G**) Proportion of the synchronous and asynchronous component. (**H**) Recovery pulse, measured as the charge of a single pulse given 2 s after the 5 Hz train relative to the charge of the 1^st^ EPSC of the train. (**I-Q**) Rundown during repetitive stimulation of 100 APs at 40 Hz. (**I**) Representative EPSC trace (top) showing asynchronous current build-up (grey shading) in control neurons and enlarged first ten EPSCs from all groups (bottom). Dashed lines represent the baseline and the delimitation between synchronous and asynchronous release^7^. (**J**) Absolute total charge rundown together with absolute amplitude for the first five AP (inset). (**K**) Normalized total charge. (**L**) Normalized asynchronous release. (**M**) Absolute total charge and (**N**) Normalized total charge from the entire train. (**O**) Proportion of the synchronous and asynchronous component. (**P**) Recovery pulse after a 40 Hz train. (**Q**) Cumulative synchronous charge from 40 Hz burst and linear extrapolation from the 40 last APs giving an estimate of the RRP size (**R**). (**S**) Release probability, calculated from the 1^st^ EPSC charge ratio and the RRP size charge. Data are represented as mean ± SEM; Kruskall Wallis ANOVA and one way repeated ANOVA, Pairwise Post-hoc tests (*p < 0.05, **p < 0.01, ***p < 0.005). Red symbols indicate outliers. For each group, the number of recordings (n) and the number of independent experiments (N) are indicated as “n/N”^66^.

To investigate further how sustained evoked release was affected by Doc2b^DN^ and Doc2b^6A^ in DKO neurons, we performed repetitive stimulation with 100 APs at low (5 Hz) and high (40 Hz) frequency (Fig. 7). Overexpression of Doc2b^DN^ and Doc2b^6A^ caused a similar fast depression of the EPSC charge, both at 5 Hz (Fig. 7A–C) and 40 Hz (Fig. 7I–K) without altering the total charge transfer (Fig. 7E,M). A particularly steep rundown was observed in mutant-expressing neurons during the first five EPSCs (top plots in Fig. 7B,J). Both mutants caused a significant decrease in the cumulative normalized charge (Fig. 7F,N). This phenotype was more evident for normalized than for absolute EPSC charges (compare Fig. 7C–F,K–N). In contrast to mutants, overexpression of wildtype Doc2b did not cause any significant change in synaptic depression compared to control (Fig. 7B–F,J–N).

During repetitive stimulation, a rundown of synchronous release is accompanied by an increased asynchronous release component. In view of previous observations implicating Doc2 proteins in asynchronous release^32^, we tested if Doc2b overexpression affects the proportion of synchronous and asynchronous components to the total EPSC charge (Fig. 7G,O), quantitated as previously described^7^.

During repetitive stimulation at 5 Hz, the expression of mutant Doc2b did not affect the balance between synchronous and asynchronous release (Fig. 7G). However, wildtype Doc2b significantly affected the release balance in favor of asynchronous release. Note that the asynchronous component is generally small for low frequency stimulation. In contrast, during 40 Hz stimulation, the balance between synchronous and asynchronous was unaffected for all groups. To assess the rate of synaptic recovery after intense stimulation (Fig. 7H,P), we recorded the EPSC from a single AP, triggered 2 seconds after the end of a 5 or 40 Hz train and compared it to the 1^st^ EPSC from the burst. Interestingly, cells expressing wildtype Doc2b displayed a potentiation of the recovery pulse compared to control group for both 5 Hz and 40 Hz trains (Fig. 7H,P). Doc2b^WT^ expressing cells showed 67 ± 3.5% recovery, whereas Doc2b^DN^ (50 ± 1.9%) and Doc2b^6A^ expressing cells (48 ± 1.8%) recovered more slowly to similar levels as GFP-expressing control group (52 ± 2.5%). These data suggest that wildtype Doc2b contributes to synaptic recovery in a manner that requires Ca^2+^ binding.

The ready-releasable-pool (RRP) size, calculated by linear interpolation of the cumulative synchronous release from 40 Hz trains (Fig. 7Q,R), appeared unchanged by expression of Doc2b^WT^ or mutants (Fig. 7Q,R) but the resulting Pvr was significantly raised by Doc2b^6A^ (Fig. 7S).

To clarify whether Doc2b mutant phenotypes on evoked release were dominant, we reproduced the repetitive stimulation experiment in WT neurons (Fig. S4). As in DKO neurons, WT cells expressing mutants showed faster synaptic depression accompanied by reduced cumulative normalized release (Fig. S4G,J,N,Q). Doc2b^WT^ potentiated the EPSC recovery and this effect was absent in mutants (Fig. S4H,S). The RRP size was similar for all groups and the release probability was significantly increased in both Doc2b^DN^ and ^6A^ expressing neurons compared to control and Doc2b^WT^ groups (Fig. S4T,U), confirming the findings in DKO neurons. Together, we conclude that both mutants affect short term plasticity in paired pulse and repetitive stimulations. Moreover, the mutations are dominant as they affect neurotransmitter release in both WT and DKO neurons.

## Discussion

To shed light on Doc2b protein function in synaptic release, we studied the Ca^2+^ binding site mutants Doc2b^DN^ and Doc2b^6A^. We found that both mutants (i) mimic an activated state at low [Ca^2+^] resulting in constitutive membrane enrichment and increased spontaneous release rates; (ii) induce faster depression during repetitive neuronal stimulation (paired pulse, train stimulation, recovery after repetitive stimulation).

In previous studies, Doc2b^DN^ has been interpreted as a gain-of-function mutant based on the increased phospholipid binding at rest (i.e. the mutations were considered to mimic Ca^2+^ binding). Doc2b^6A^ was described to be a loss-of-function mutant based on the loss of Ca^2+^ binding capacity. This classification as gain- and loss-of-function mutants had important implications: the enhanced spontaneous release in Doc2b^DN^ overexpression was taken as evidence that Doc2b functions as a Ca^2+^ sensor^21^, while the very similar phenotype in Doc2b^6A^ overexpressing neurons was taken to demonstrate a Ca^2+^-independent function^29^.

Our data demonstrate that Doc2b^6A^ shows constitutive membrane binding, as supported by other studies^27,43^. Thus, the Doc2b^6A^ is not a loss-of-function mutant. At the same time however, the Doc2b^DN^ mutant is also not a pure gain-of-function mutant, as indicated by the incapacity to potentiate release after repetitive neuronal activity. In fact, both mutants share very similar phenotypes in enhancing lipid and membrane association, neurotransmitter release at rest and during stimulation but also impaired EPSC potentiation after intense stimulation. The dual effect of Ca^2+^ binding site mutations could be attributed to the different surface charge distribution of the aspartates in the Ca^2+^- and membrane binding site of the C_2_ domains. In Doc2b wildtype protein, the aspartates residues are neutralized by binding of Ca^2+^ ions. In the mutants, the neutralization of the aspartates could support membrane binding at rest, but this binding may not reach the same affinity as with Ca^2+^-bound aspartates. Alternatively, the mutations could slightly misplace the membrane-inserting residues in the loops surrounding the aspartates.

The Ca^2+^-dependent membrane association of wildtype and mutants seems slightly more complex than might be expected. At high (>500 nM) [Ca^2+^]_free,_ isolated C_2_AB fragments showed a loss of liposome clustering activity using synthetic membranes composed of 75% DOPC and 25%DOPS. Recent investigations, revealed a similar effect for the Doc2b^6A^ mutant (termed 6x) as its liposome binding capacity dropped in presence of Ca^2+^ ^27^. In our hands a similar reduction in lipid binding occurred at high (>700 nM) [Ca^2+^]_free_ for Doc2b^WT^-C_2_AB. Syt-7 was also reported to have a reduced lipid binding activity at high [Ca^2+^]_free_, effect that was associated with an inhibitory effect on norepinephrine secretion in PC12 cells^54^. Another similar observation is the negative effect of high [Ca^2+^]_free_ on Syt-1 *in vitro* fusion ability^55^, together suggesting that most Ca^2+^-sensors function in a limited [Ca^2+^] window and that higher [Ca^2+^] might have a negative effect.

In a previous study using isothermal titration calorimetry, the D218,220N mutation did not completely abrogate Ca^2+^ binding activity in a recombinant C_2_AB fragment named C_2_A_CLM_B (for calcium ligand mutant)^44^ likely due to its functional C_2_B domain. In our hands, Doc2b^DN^ specifically shifted the synchronous/asynchronous release balance in favor of fast release (Fig. S4R). This could be explained by the constitutive localization to the PM and the reduced Ca^2+^-sensitivity of this mutant. Moreover, C_2_A_CLM_B_CLM_ carrying the additional mutation D357,359N in the C_2_B domain abolished Ca^2+^ association to C_2_ domains, suggesting that in the absence of lipids, the C_2_B domain is solely responsible for the Ca^2+^ binding activity^44^. The C_2_A_CLM_B_CLM_ mutant also greatly increased spontaneous release.

In presence of phospholipids, the negatively charged head groups may stabilize bound Ca^2+^ ions and the apparent Ca^2+^ affinity may be higher^56,57^. D220N substitution within the C_2_A Ca^2+^-binding-pocket of Doc2b, alone or in combination with other mutations is responsible for the constitutive membrane binding^47^. A single residue substitution (D303N) completely abolishes Doc2b translocation and this mutant does not rescue spontaneous release^27^, confirming the idea that Doc2b acts as a Ca^2+^-sensor. Considering all these mutants, there is a striking correlation between the Ca^2+^-dependent phospholipid association of Doc2b under resting [Ca^2+^] and its function in spontaneous neurotransmission.

Our results show that Doc2b^DN^ and ^6A^ mutants affect synaptic plasticity during repetitive activity. In chromaffin granule secretion, wildtype Doc2b serves both positive and negative roles^43^, a feature that is shared with other exocytotic proteins such as synaptotagmins, complexins and munc18s. In this system, Doc2b^DN^ and Doc2b^6A^ favour immediate chromaffin granule fusion but impair sustained release at high [Ca^2+^]. This phenotype might also be a collateral effect of constant vesicle fusion at rest, exhausting the immediate releasable pool (IRP). In WT neurons, we also observed a modification of first evoked release by Ca^2+^ binding mutants, but this effect was not significant in Doc2a,b DKO neurons. Despite the alteration of several synaptic release parameters by Doc2b mutants, the overexpression of Doc2b^WT^ (Figs 6, 7 and S4) or the removal of endogenous Doc2b^21^ does not importantly affect evoked release, suggesting that this is not a major function of Doc2b in synapses. The effect of Doc2b mutants on evoked release in WT neurons may possibly expose an ectopic function under experimental conditions.

On the other hand, Doc2b^WT^ overexpression and rescue revealed a consistent effect in post-burst recovery (Figs 7H,P and S4L,S), absent in Doc2b^DN^ and Doc2b^6A^ expressing neurons. An effect of Doc2b^WT^ in post-tetanic stimulation has already been reported^40^. Both findings support a role for Doc2b in superpriming probably related to high residual [Ca^2+^] after burst activity. The ineffectiveness of Doc2b^DN^ and Doc2b^6A^ in post-burst recovery might result from the lack of their ability to translocate in response to Ca^2+^ elevation, suggesting that wildtype Doc2b contributes to synaptic recovery in a manner that requires Ca^2+^ binding. In Syt-1 KO neurons the translocation-impaired Doc2b^D303N^ mutant also abolished asynchronous release enhancement^47^, revealing a correlation between translocation ability and neurotransmitter release enhancement, thus consolidating the hypothesis that translocation is necessary for release potentiation.

Additionally, a subtle but significant shift in favour of asynchronous release during repetitive stimulation appeared in DKO neurons rescued with Doc2b^WT^ (Fig. 7G) but not with mutants. During intense release, as suggested by the reduced *in vitro* lipid binding at high [Ca^2+^], Doc2b^WT^ could inhibit fast exocytosis acting as a clamp of the fast fusion machinery. Our data support the idea that wildtype Doc2b spares the ready releaseable pool (RRP) and changes the balance in vesicle recruitment from a reserve pool as recently claimed in a peer investigation^43^.

Doc2b^DN^ and ^6A^ mutant overexpression caused an increase in the spontaneous release frequency and short-term depression during trains and paired pulse stimulation. These effects may be attributable to a change in the vesicular release probability (Pvr), an idea supported by Figs 7S and S4U. Alternatively however, changes in the short-term plasticity can also be shaped by altered vesicle recruitment from the reserve pool to the RRP, including ultrafast recruitment of reluctant SVs to release sites during repetitive stimulation^58^. When SV recruitment is reduced, short-term facilitation will turn into depression^59^. Molecular perturbations of Ca^2+^-sensors like Syt-1 can lead to labile primed states affecting short-term plasticity^60^. Therefore, an alternative explanation for the fast rundown would be that Doc2b mutants induce labile docking of newly recruited SVs, causing RRP depletion and synaptic fatigue.

In our hands, Doc2b^DN^ and Doc2b^6A^ did not enhance delayed release, as was previously observed for another Ca^2+^-ligand mutant (D218,220,357,359N) in locally stimulated neuronal networks^44,47^. Also, we did not observe increased RRP sizes in presence of Doc2b^DN^ and ^6A^ which were reported for that mutant^44^. These differences could be explained by different cell culture conditions, stimulation method or by the different point mutations used. However, our results are compatible with a study in Syt-1 KO neurons^47^ where Doc2b Ca^2+^-binding site mutants enhanced the remaining asynchronous release component triggered by a single AP.

We conclude that the Doc2b^DN^ and Doc2b^6A^ mutants do not represent divergent gain- and loss-of-function mutants but show similar behavior, characterized by increased neurotransmitter release at rest and during the early phase of neuronal activity. Moreover, the strict correlation for the wildtype protein between plasma membrane association and spontaneous release frequency supports a direct role as a Ca^2+^ sensor. Our results provide more insight in the various functional properties of Doc2b and its Ca^2+^ binding site mutants, which fit published conflicting data.

## Material and Methods

### Mouse lines

Animals were housed, bred and handled in accordance with Dutch and EU governmental guidelines. Protocols were approved by the VU University Animal Ethics and Welfare Committee (approval number FGA 11-06). Wildtype C57BL/6J mice were obtained from Charles River Laboratories. Doc2 a & b double knockout mice (DKO), maintained on the same C57BL/6J genetic background, were previously described^21^. To dissociate brain tissue from DKO mice, hippocampi were isolated at postnatal day 1 (P1). For wildtype mice, E18–stage embryos were used. In this case, pregnant females were sacrificed by cervical dislocation, embryos were obtained by caesarian section, decapitated and used for dissection.

### Primary culture of mouse neurons

To isolate mouse neurons, brains were placed in Hanks buffered salt solution (HBSS, Sigma) buffered with 1 mM HEPES (Invitrogen). After meninges removal, hippocampi and cortices were dissociated and separately treated. The tissue was incubated with 0.25% trypsin (Invitrogen) for 20 min at 37 °C and washed in DMEM. Cells were dissociated by trituration with a fire-polished Pasteur pipette and counted in a Fuchs‐Rosenthal chamber. Neurons were plated in warmed Neurobasal medium supplemented with 2% B-27, 1.8% 1 M HEPES, 0.25% glutamax and 0.1% Pen-strep (all products Invitrogen) as previously established^61^.

Electrophysiology experiments were performed in network or autaptic cultures. For network cultures, hippocampal neurons were plated at a density of 25K cells per well in 12-wells plates on etched glass coverslips containing a confluent layer of rat astrocytes^61^. For autaptic cultures, 1.5K cells per 12-well or 3K per 6-well were plated on coverslips with astrocyte micro-islands stamps^61^. For live imaging, hippocampal neurons were plated on rat astrocytes in low density networks (10K per 12-well)^61^. For western blotting, cortical neurons were plated at 300K per well in 6-well plates without coverslips, coated overnight with 0.0005% poly-L-Ornithine (Sigma) and 2 µg/ml laminin (Sigma) in PBS and washed with sterile water.

### Viral overexpression of Doc2b

For functional assays, Doc2b and EGFP were expressed as separate proteins from a single mRNA using an IRES2 internal ribosome entry site. Wildtype rat Doc2b^WT^ (LIP#1984) was compared to Doc2b^DN^ carrying the D218, 220N mutation (LIP#1985)^21,41^ and Doc2b^6A^ carrying the D163, 218, 220, 303, 357, 359A mutation (LIP#1986)^29^. Lentiviral infectious particles were packaged in HEK293T human embryonic kidney cells with a passage number lower than 25, maintained in Dulbecco’s Modified Eagle Medium (DMEM) supplemented with 10% fetal calf serum, 50 U/ml penicillin-streptomycin and 1x non-essential amino acids (Gibco). At 2 days *in vitro* (DIV2) the cells were transfected at 50% confluence with three plasmids: p.MDG2 (encoding the viral envelope protein), pCMVΔR8.2 (encoding packaging factors) and a p156RRL-derived plasmid encoding Doc2b. LIP#1984 encoded Doc2b^WT^, LIP#1985 Doc2b^DN^ and LIP#1986 Doc2b^6A^. At DIV3 the medium was changed to Optimem + 50 U/ml penicillin-streptomycin without fetal bovine serum. At DIV4, the supernatant containing infectious particles was centrifuged at 1000 × g to remove cell debris. The supernatant was concentrated by ultrafiltration using a 100 kDa cutoff membrane (UFC910024, Millipore, spun at 4000 × g for 20–30 min) to achieve a final volume of 150 µl. The LIPs were diluted to 1 ml with phosphate-buffered saline, filtered through 0.45 µm and stored in aliquots at −80 °C until use. Neurons were infected at DIV1 to induce Doc2b expression. To investigate subcellular protein localization, Doc2b was C-terminally tagged with EGFP. Neurons were transduced with Semliki infectious particles 10 to 12 hours before experimentation (SIP#293 encoding Doc2b^WT^, SIP#244 encoding Doc2b^DN^ and SIP#295 encoding Doc2b^6A^) as described^43^.

### Electrophysiology in primary hippocampal neuronal networks and autapses

For electrophysiology, both continental and island cells were used between DIV 14 to 21. Doc2b-expressing cells were identified by monitoring EGFP fluorescence. The standard extracellular medium included 140 mM NaCl, 2.4 mM KCl, 4 mM CaCl_2_, 4 mM MgCl_2_, 10 mM HEPES, 10 mM glucose, 300 mOsm, pH 7.3. Our standard intracellular (patch pipette) solution was EGTA free to prevent Ca^2+^ buffering; it constituted 125 mM K-gluconate, 10 mM NaCl, 4.6 mM MgCl, 4 mM K_2_-ATP, 15 mM creatine phosphate and 10 U/ml phosphocreatine kinase, 300 mOsm, pH 7.3. To record spontaneous excitatory events in network cultures, 1 µM tetrodotoxin (TTX, Abcam) and 20 µM gabazine (Sigma Aldrich) were added to the extracellular medium. In autapses, only gabazine was added. Where indicated, the Ca^2+^ ionophore calcimycin (A23187, Sigma) was used at a final concentration of 10 µM and applied by puff for 100 seconds through a barrel placed in the vicinity of the soma.

The patch pipettes were made of borosilicate and pulled using a multi-step filament pulling (P-1000, Sutter Instruments, Novato, USA) to achieve a pipette resistance of 3 to 5 MOhm. In whole-cell configuration, neurons were voltage clamped at −70 mV with an Axopatch 200B or Multiclamp 700B amplifier (Molecular Devices). Signal was low-pass filtered at 1 kHz and digitized at 10 kHz with a Digidata 1440 A or 1550 (Molecular Devices). Neurons with a series resistance (R_s_) exceeding 15 MOhm or with an R_s_ increase beyond 20% of the initial value were excluded. R_s_ was compensated to 70%. EPSCs were elicited by depolarizing the cell to 0 mV for 1 ms. Standard stimulation paradigms comprised spontaneous activity recording, paired pulse stimuli with intervals from 20 ms to 1 s, two trains of each 100 action potentials at 5 Hz and 40 Hz. Each train was followed by a single stimulus at 2 s after the last depolarization to test synaptic recovery.

Miniature EPSCs (mEPSCs) were detected using Mini Analysis 6.0 (Synaptosoft Inc.), using thresholds of 7 pA for event amplitude and 15 pC for area. Evoked release events (paired pulse stimulations) were analyzed using an in-house routine in the MATLAB^®^ (Mathworks) environment^62^ to calculate the paired pulse ratio, the EPSCs charge and amplitude. The paired-pulse ratio was calculated by dividing the 2^nd^ EPSC from paired response to the 1^st^ EPSC. The 1^st^ EPSC quantal content was estimated by dividing the mEPSC charge by the single EPSC charge from the same cell. Total, synchronous and asynchronous EPSC charge components were calculated using a homemade Matlab^®^ routine. Our program uses 2 different baselines: first a straight line connecting the current before the train to the baseline after full recovery. This baseline is used to calculate the total charge. To separate the synchronous and asynchronous charge components, the second baseline connects the EPSC values immediately before each pulse following a previously described method^7^. Both components were calculated using cubic interpolation. To calculate the pool size, the cumulative synchronous charge was used. In order to determine the Y-intercept, data were fitted in their entirety by an exponential and a linear component in the form of the function beside ${\rm{F}}({\rm{x}})=({\rm{A}}\,\ast \,(1-{{\rm{e}}}^{-\frac{{\rm{x}}}{{\rm{tau}}}}))+({\rm{a}}\,\ast \,{\rm{x}}+{\rm{b}})$. The pool size was determined by summing the A and b parameter from the fitting function. The release probability (Pvr) was then obtained by the ratio of the single EPSCs charge on the total RRP size. The EPSC recovery was calculated by the ratio of a single EPSC charge triggered 2 s after the end of each train to the first EPSC charge from that train. Calcimycin evoked responses were quantitated using Clampfit 10.4 (Molecular Devices) by measuring the total charge transfer during the compound application.

### Doc2b live microscopy

For Doc2b protein translocation imaging, hippocampal neurons from wildtype mouse at embryonal day E18 were dissociated and platted at 10 K per well on glia layer. Cells were double-infected with Semliki virus encoding for Doc2b^WT^-EGFP and either Doc2b^DN^-mCherry or Doc2b^6A^-mCherry at DIV15. The two SIP stocks were first mixed in a 1:1 volume ratio and then added to each coverslip. Live imaging was performed 8–11 h post infection using a Nikon A1R confocal laser microscope controlled by NIS-elements AR software version 4.30 (Laboratory Imaging). Extracellular solutions for the chamber perfusion was similar to electrophysiology experiments. Neurons were stimulated after 15 seconds of rest by puff application for 30 seconds of a depolarizing solution containing 82.4 mM NaCl, 60 mM KCl, 4 mM CaCl_2_, 4 mM MgCl_2_, 10 mM HEPES, 10 mM glucose, 300 mOsm, pH 7.3. The puff was triggered from a Master 8 connected to a valve opening system (WPI type A385). ImageJ was used for data analysis. First, 2 to 6 ROIs depending on cell size and morphology were drawn as lines with a thickness of 10 pixels spanning a neurite or soma. Line scans were obtained from the 4 first stacks each in the resting condition (Fig. 2B,C,G,H; “naïve”) and the KCl condition. The fluorescence intensity in regions corresponding to the PM and cytosol were used to calculate the PM/C ratio for each ROI.

### Solutions for phospholipid-binding assays

Chelated Ca^2+^/EGTA solutions containing 50 mM HEPES, pH 7.4, 100 mM KCl, and varying concentrations of Na_2_EGTA and CaCl_2_ (0 to 10 mM) were made by predicting [Ca^2+^]_free_ with MaxChelator (http://maxchelator.stanford.edu/CaEGTA-TS.htm). Actual [Ca^2+^]_free_ was verified in each solution by recording fluorescence excitation spectra of fura-2 (Invitrogen, 0.07 µM) at an emission wavelength of 510 nm on a LS55 fluorescence spectrophotometer (Perkin Elmer). [Ca^2+^]_free_ was calculated as Kd × [(R − R_min_)/(R_max_ − R)] × (F_max_^380^/F_min_^380^), where F^380^ is the fluorescence intensity at ʎ_excit_. = 380 nm and R is the ratio F^340^/F^380^. Kd_EGTA_ was measured at 34.906 nM.

Liposomes were formed by drying chloroform solutions containing 25% 1,2-dioleoyl-*sn-*glycero-3-phospho-L-serine (DOPS, Avanti Polar Lipids) and 75% 1,2-dioleoyl-*sn*-glycero-3-phosphocholine (DOPC, Avanti Polar Lipids)under a nitrogen stream. The phospholipids were resuspended in 50 mM HEPES, 100 mM KCl, pH 7.4 to a final concentration of 1 mg/ml, sonicated 5 times for 10 seconds and centrifuged for 90 min at 21,000 × g to clear the liposomes from large aggregates as described previously^48^.

### Expression and purification of Doc2b C_2_A and C_2_AB fragments

The C_2_A (aa125-255) and C_2_AB (115-412) fragment of Doc2b^WT^, Doc2b^DN^ and Doc2b^6A^ were expressed as glutathione-S-transferase (GST) fusion proteins in the E. coli strain BL21 and purified as described^63^. GST-C_2_A was eluted from glutathione-agarose beads by glutathione, leaving the GST tag attached and allowing GST dimerization. Each C_2_AB batch was incubated with 10 U thrombin (Sigma, T6884) diluted in 200 µl of thrombin elution buffer (50 mM Tris, 150 mM NaCl, 2 mM DTT) at 4 °C for 16 h, thus removing the GST tag (see Supplementary Fig. S1). In both cases, Ca^2+^-dependent C_2_-membrane interaction causes liposome aggregation which can be measured by an OD 350 nm increase^48,64^. Protein amounts, potential contamination or degradation were verified by SDS gel electrophoresis. To compare lipid-binding activities, recombinant proteins were pooled from the following number of expression cultures: 4 for C_2_A^WT^, 5 for C_2_A^DN^, 5 for C_2_A^6A^, 3 for C_2_AB^WT^, 6 for C_2_AB^DN^, 6 for C_2_AB^6A^.

### Phospholipid-binding assays

To measure phospholipid binding, 20 µl of liposomes were mixed with 78, 70, 70 µl for GST-C_2_A^WT^, GST-C_2_A^DN^, GST-C_2_A^3A^ respectively and 73, 70, 60 µl for C_2_AB^WT^, C_2_AB^DN^, C_2_AB^6A^ respectively of buffered Ca^2+^/EGTA solution in a quartz cuvette to a final concentration of 0.5 mg/ml and the absorption at 350 nm was monitored for 10 minutes at 0.2 s intervals in a Cary 50 UV-Vis spectrophotometer. GST-C_2_A and C_2_AB protein used concentration were determined by measurement of their lipids aggregation capacity to reach a maximal OD 350 nm around 0.5 and measured afterward by SDS-PAGE. After 60 s of baseline recording, GST-C_2_A or C_2_AB protein was added to a final concentration of 9 µM, 26 µM, 24 µM for GST-C_2_A^WT^, GST-C_2_A^DN^, GST-C_2_A^3A^ respectively and 0.36 µM, 0.39 µM, 0.9 µM C_2_AB^WT^, C_2_AB^DN^, C_2_AB^6A^ respectively, inducing liposome binding and consequently an increase of A_350_. EC50 values were manually calculated using OD 350 nm half-maximum X-intercept from raw data.

### Immunostaining and confocal imaging for synapse counting

After lentiviral expression, hippocampal neurons expressing Doc2b^WT^, Doc2b^DN^ or Doc2b^6A^, as marked by IRES-eGFP fluorescence, were fixed for 20 minutes at RT in 3.7% paraformaldehyde. After washing with PBS, cells were permeated with 0.5% Triton X-100 for 5 minutes and incubated for 20 minutes with 2% normal goat serum and 0.1% Triton X-100 to prevent aspecific binding. Coverslips were incubated for 2 hours at RT or overnight at 4 °C in presence of polyclonal chicken anti-MAP2 (Abcam, ab5392) and polyclonal guinea pig anti-Synaptophysin1 (SySy, 101004) both diluted 1000-fold. After washing, cells were incubated overnight at 4 °C with Alexa-546- and -647-conjugated secondary antibodies (1:1000, Invitrogen), washed again and mounted with Mowiol.

Images were acquired with a confocal microscope LSM 510 (Carl Zeiss) with 488 nm, 543 nm, 633 nm lasers, using a 40x oil immersion objective and a scan resolution of 1024 × 1024 pixels. Stacks of images with an optical thickness of 0.4 µm were obtained. Neuronal morphology characteristics were analyzed with an automated image analysis MATLAB^®^ (Mathworks) routine^65^.

### Western blot

Cortical neuron cultures from WT and Doc2a, b DKO mice were plated to 200 K cells per well, infected at DIV 1 with lentiviral Doc2b constructs (LIP #1984, #1985, #1986 as above) and harvested at DIV 17. Cells were washed 2 times with PBS, lysed in Laemmli sample buffer, loaded with 50% or the totality of each well for WT and DKO cells respectively, separated by SDS-PAGE and blotted on PVDF membrane (Biorad). Membranes were blocked in 2% skim milk powder (Merck) and 0.5% FCS (Gibco) in PBS with 0.01% Tween-20 (Sigma-Aldrich). Doc2B polyclonal antibody 13.2 was used as primary antibody (1:500) for incubation overnight at 4 °C. Goat anti rabbit alkaline phosphatase (Jackson lab) was used as secondary and Attophos (Promega) as substrate for 30 min incubation at RT. Reprobing was made for actin immunostaining with monoclonal anti-actin antibody C4 (1:3000; Chemicon) and Goat anti mouse alkaline phosphatase as secondary Ab (1:10000).

### Statistical analysis

All statistical analysis was performed using SPSS v.25.00 (IBM Corp., Armonk, NY, USA). All data are reported as mean ±SEM, except when specified. The number of measurements “n”, indicate the number of cells per group and “N” the number of independent observations, meaning the number of experimental weeks. Data were checked for normality using Shapiro-Wilk and Kolmogorov-Smirnov tests. Homogeneity of the variance was assessed with Levene’s test and the sphericity assumption was tested with Mauchly’s test. Parametric or non-parametric tests were run depending on homogeneity assumption respect or violation. Moreover, if the sphericity assumption was not met the Greenhouse-Geisser correction was used to adjust the degree of freedom. Considering that groups were independently acquired from one another, independent samples tests were performed: i) For two groups a Mann-Whitney U test was performed; ii) For more than 2 groups, one-way repeated measure ANOVA or Friedman’s ANOVA or Kruskall-Wallis test were used followed by pairwise post-hoc. For each experiment, the p-value alpha significance threshold was adjusted for multiple testing. The effect size was calculated for the independent samples Mann-Whitney U test as ${\rm{r}}={\rm{Z}}/\sqrt{{\rm{N}}}$, for one-way repeated ANOVA and Friedman ANOVA as ${\rm{r}}=\sqrt{{{\rm{\chi }}}^{2}/({\rm{N}}+{{\rm{\chi }}}^{2})}$and for Kruskall-Wallis test as ${\rm{r}}={\rm{Z}}/\sqrt{{\rm{N}}}$. P-values lower than the accepted α-significance were highlighted in bold and the effect size was reported. Post-hoc tests were all pairwise comparison. In graphs, the empty dots represent single data points, bar plots represent the mean and error bars the SEM. Statistical outliers are depicted by red ‘stars’ symbols.

## Supplementary information

Supplementary Information

## Data Availability

The datasets generated and analysed during the current study deposited in the VU Institutional Research Data Management system (Quentin T. Bourgeois; Matthijs Verhage; Alexander J. Groffen, “Replication Data for Doc2b Ca2+ binding site mutants enhance synaptic release at rest at the expense of sustained synaptic strength”, https://hdl.handle.net/10411/I6Q2G2, DataverseNL).
