# Supplementary material for: Doc2b Ca2+ binding site mutants enhance synaptic release at rest at the expense of sustained synaptic strength
Source: Sci Rep. 2019 Oct 8;9:14408. doi: 10.1038/s41598-019-50684-1 (PMC6783474; doi:10.1038/s41598-019-50684-1)
Supplement: Supplementary file 1 — Supplementary Information [file 41598_2019_50684_MOESM1_ESM.pdf]

Supplementary Information

Title:

Doc2b Ca^{2+} binding site mutants enhance synaptic release at rest at the expense of sustained synaptic strength

Authors:

Quentin Bourgeois-Jaarsma[1], Matthijs Verhage[1,2], Alexander J. Groffen[1,2*]

Contents:

Supplementary figures S1 – S5

Supplementary Table 1

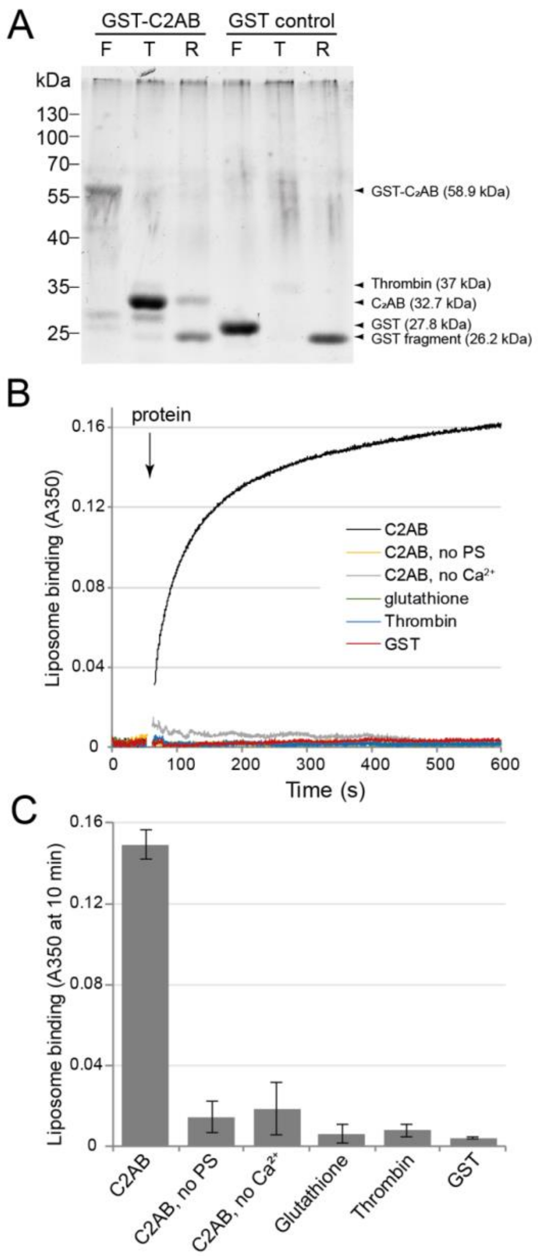

Supplementary Figure S1: Liposome binding by GST fusion proteins depends on PS and Ca^{2+} and is not attributable to GST, thrombin or glutathione. (**A**) SDS-PAGE analysis of recombinant GST-C2AB or GST alone expressed in E.coli. F indicates full-length fusion protein, eluted from glutathione-Sepharose beads with 5 mM soluble glutathione; T indicates a protein fragment eluted from the beads by proteolytic cleavage with thrombin; R indicates the residual GST fragment remaining on the beads after thrombin cleavage, subsequently eluted with 5 mM glutathione. Predicted molecular weights are indicated at the right. (**B**) Kinetic traces of phospholipid binding, quantitated as absorption at 350 nm in presence of 834 nM $[Ca^{2+}]_{free}$ and liposomes containing DOPC/DOPS (80/20) unless indicated otherwise. After 60 s (arrow) the indicated sample was added: C2AB (50 µl of fraction 'T' shown in panel A), GST (50 µl of fraction 'F'), thrombin (50 µl containing 2.5U, equal amount as in fractions 'T'), glutathione (50 µl of 5 mM glutathione, equal as in fractions 'F'). For the 'no PS' control, 100% DOPC liposomes were used. For the 'no Ca^{2+}' control, a buffer containing 10 mM EGTA was used. (**C**) As a measure for total phospholipid binding, the A350 from the last 20 s was averaged. Graph represents mean ± sem of 2-4 assays (C2AB: n=4; others: n=2).

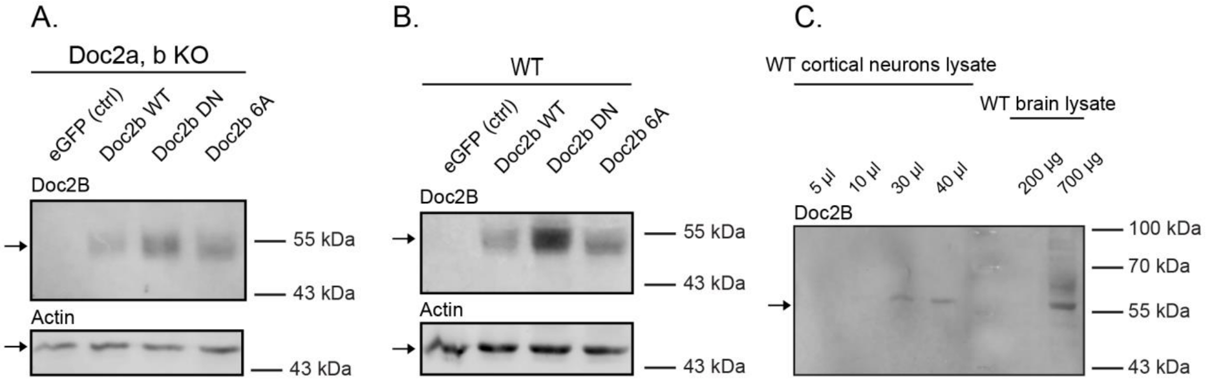

Supplementary Figure S2. Doc2b expression levels in wildtype and Doc2a,b double knockout (DKO) neurons. Western blots from (**A**) DKO and (**B**) WT high density neuron cultures overexpressing wildtype or mutant Doc2b confirming the overexpression of Doc2b^{WT} and mutants by lentiviral particles. Each lane contains the lysate from 100K cortical cells transduced at 1 DIV and harvested at 17 DIV. (**C**) Endogenous Doc2b could not be detected in standard conditions but became detectable by loading larger sample volumes of cortex or total brain lysate (µg indicates total wet weight of brain tissue). Doc2b immunoreactivity in overexpressing neurons was higher compared to endogenous levels in non-transfected wildtype neurons. For cropped blots, full-length blots are available in the replication dataset as detailed in the Data Availability Statement.

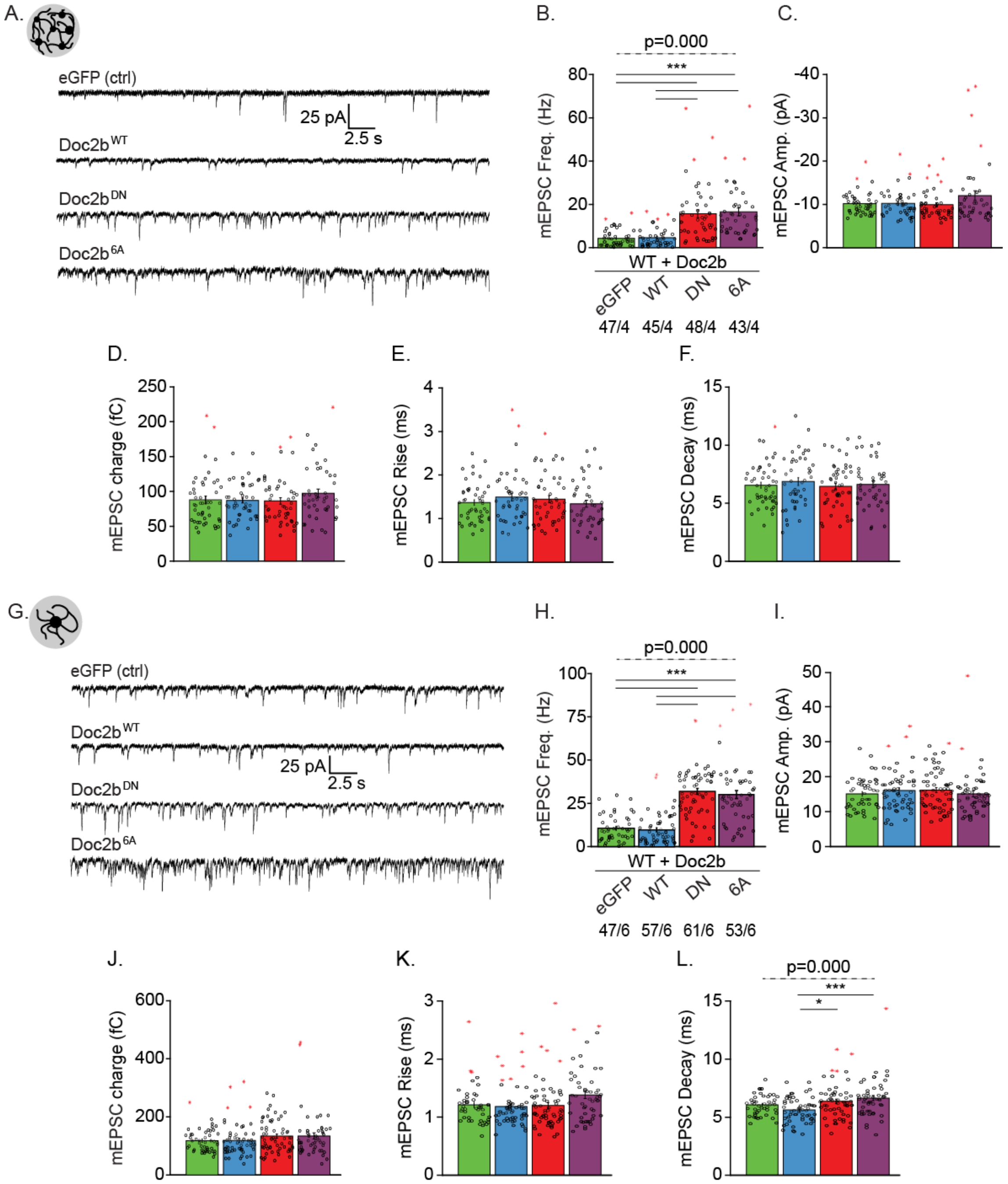

Supplementary Figure S3. Doc2b^{DN} and Doc2b^{6A} mutations enhance the frequency of spontaneous release in wildtype hippocampal neurons. (**A**) Representative mEPSC recordings in wildtype neurons overexpressing eGFP (ctrl), Doc2b^{WT} or mutants (Doc2b^{DN}, Doc2b^{6A}), cultured in networks in presence of 1 µM TTX and 20 µM gabazine. (**B**) Quantification of spontaneous neurotransmitter release frequency and (**C-F**) amplitude, charge, rise and decay time. (**G**) Representative recordings in isolated neurons cultured on microglial islands (autapses). (**H-L**) Quantification of the frequency and postsynaptic parameters. Data are represented as mean ±SEM;. Kruskall Wallis ANOVA, Pairwise Post-hoc tests (***, p<0.005).). Red stars signs indicate outliers. The number of cells (n) and the number of independent experiments (N) are indicated as “n/N”.

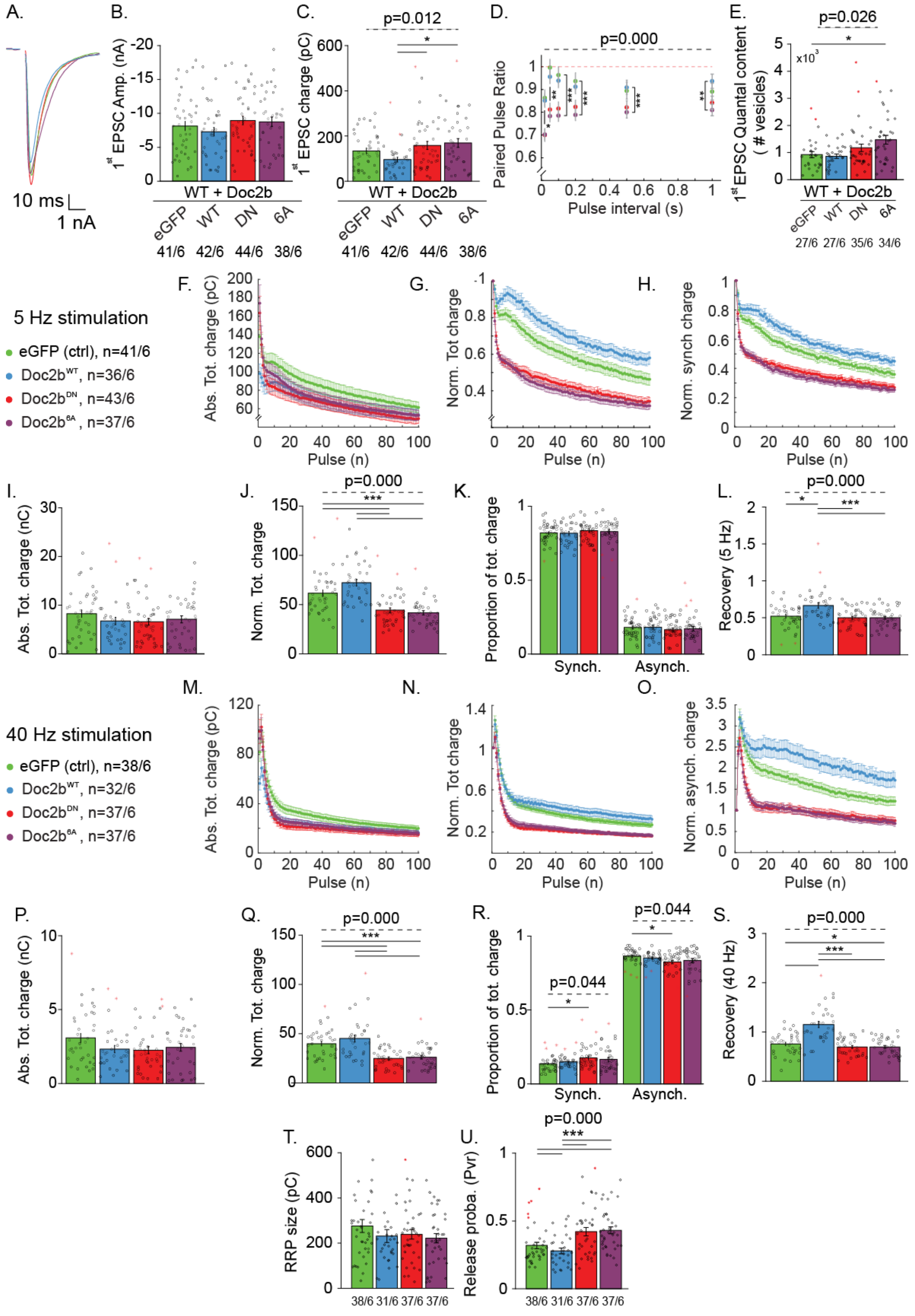
A.
10 ms
1 nA
B.
1st EPSC Amp. (nA)
WT + Doc2b
eGFP WT DN 6A
41/6 42/6 44/6 38/6
C.
p=0.012
1st EPSC charge (pC)
WT + Doc2b
eGFP WT DN 6A
41/6 42/6 44/6 38/6
D.
p=0.000
Paired Pulse Ratio
Pulse interval (s)
E.
p=0.026
1st EPSC Quantal content (# vesicles)
x10³
WT + Doc2b
eGFP WT DN 6A
27/6 27/6 35/6 34/6
5 Hz stimulation
eGFP (ctrl), n=41/6
Doc2bWT, n=36/6
Doc2bDN, n=43/6
Doc2b6A, n=37/6
F.
Abs. Tot. charge (pC)
Pulse (n)
G.
Norm. Tot charge
Pulse (n)
H.
Norm. synch charge
Pulse (n)
I.
Abs. Tot. charge (nC)
J.
p=0.000
Norm. Tot. charge
K.
Proportion of tot. charge
Synch.
Asynch.
L.
p=0.000
Recovery (5 Hz)
40 Hz stimulation
eGFP (ctrl), n=38/6
Doc2bWT, n=32/6
Doc2bDN, n=37/6
Doc2b6A, n=37/6
M.
Abs. Tot. charge (pC)
Pulse (n)
N.
Norm. Tot charge
Pulse (n)
O.
Norm. asynch. charge
Pulse (n)
P.
Abs. Tot. charge (nC)
Q.
p=0.000
Norm. Tot. charge
R.
p=0.044
p=0.044
Proportion of tot. charge
Synch.
Asynch.
S.
p=0.000
Recovery (40 Hz)
T.
RRP size (pC)
38/6 31/6 37/6 37/6
U.
p=0.000
Release proba. (Pvr)
38/6 31/6 37/6 37/6

Supplementary Figure S4. Doc2b mutants affect short term plasticity in WT autaptic neurons. (**A**) Averaged trace of a single EPSC from wildtype neurons overexpressing $Doc2b^{WT}$ and mutants. (**B**) Quantification of 1st EPSC amplitude and charge (**C**). (**D**) Paired-pulse ratio. (**E**) Single EPSC quantal content corresponding to the ratio of the EPSC charge and mEPSC charge. $Doc2b^{DN}$ and $Doc2b^{6A}$ affect vesicle release probability and thus short-term plasticity. (**F-L**) Rundown during repetitive stimulation for 100 AP at 5 Hz. (**F**) Absolute (**G**), normalized total charge (**H**) and normalized synchronous release. (**I**) Cumulated absolute and (**J**) normalized total charge from the entire train. (**K**) Proportion of the synchronous and asynchronous component. (**L**) Recovery pulse 2s after 5 Hz stimulation. (**M-S**) Rundown during repetitive stimulation for 100 AP at 40 Hz. (**M**) Absolute and (**N**) Normalized total charge. (**O**) Normalized asynchronous release. (**P**) Cumulated absolute and (**Q**) normalized total charge during the entire train. (**R**) Proportion of the synchronous and asynchronous component. (**S**) Recovery, measured after a 40 Hz train. Data are represented as mean ±SEM; Kruskall Wallis ANOVA and one way repeated ANOVA, Pairwise Post-hoc tests (*, $p<0.05$, **, $p<0.01$, ***, $p<0.005$). Red stars signs indicate outliers. The number of recordings (n) and the number of independent experiments (N) are indicated as “n/N”.

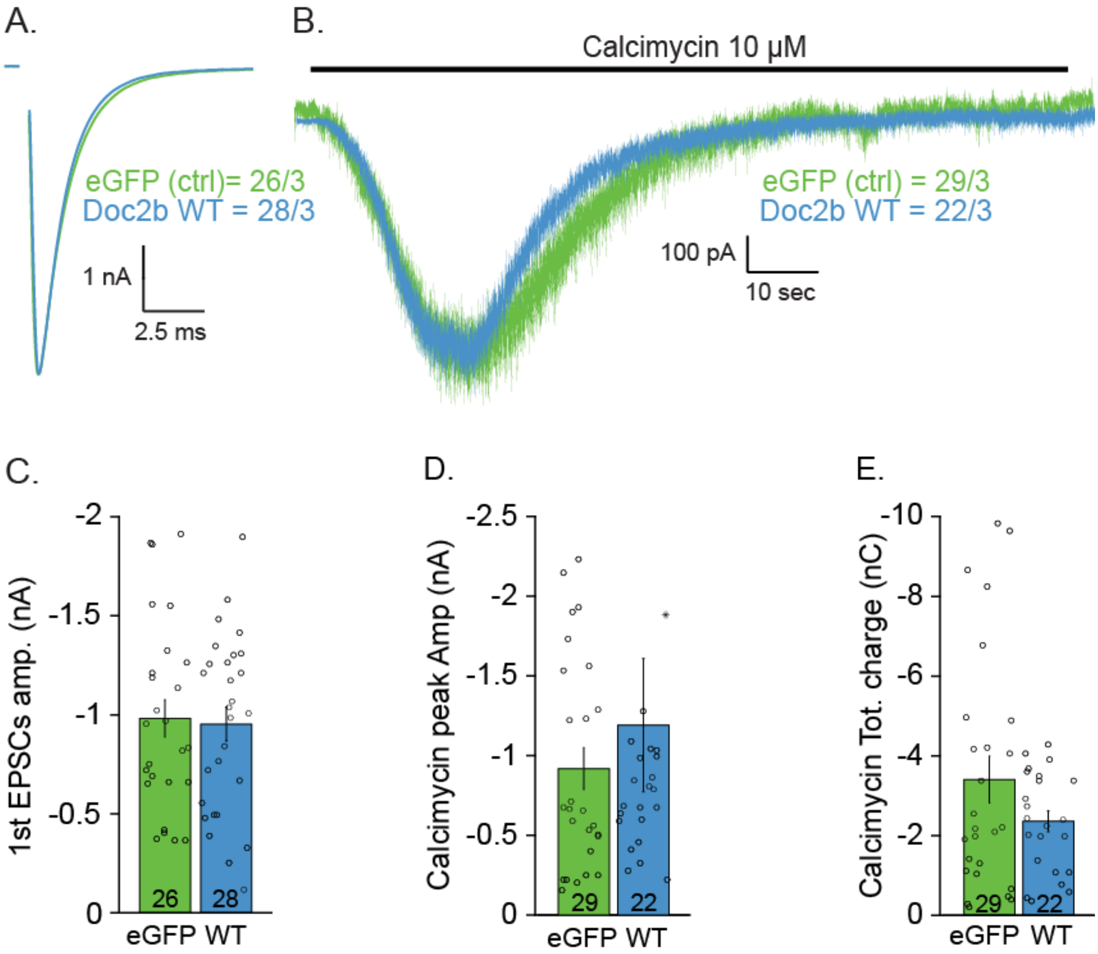

Supplementary Figure S5. Overexpression of wildtype Doc2b does not affect calcimycin-induced neurotransmitter release. Calcimycin was perfused to directly induce Ca^{2+} influx and bypass voltage-dependent Ca^{2+} channels. (**A**) Typical example of single EPSC in naïve wildtype autaptic neurons; (**B**) the EPSC induced by puff application of calcimycin for 100 s. (**C**) Quantification of EPSC amplitude. (**D**) Calcimycin-induced response peak amplitude and (**E**) total charge transfer. Data are represented as mean ±SEM. Red stars signs indicate outliers. The number of recordings (n) and the number of independent experiments (N) are indicated as “n/N”.

Supplementary Table1. Statistical analysis

Figure 2. Ca2+ binding site mutants Doc2bDN and Doc2b6A show increased plasma membrane binding under resting conditions

Figure references	Statistical test (between groups comparison)					Accepted α-significance		Sample
	(1) Friedman Test					0,05		n = 26 ROIs , N = 6 independent experiment Figure 2
	**Statistical test (within groups comparison)**					0,05		
	Pairwise comparison							
	**Parameter**	**Statistical result**	**p-value**	**Statistical test**	**Effect size (r)**	**Descriptive and pairwise comparison**		
						**groups**	**comparisons**	**Corrected p-value**
E	Ratio PM/C	$X^2(3)$=46,108	p=0,000	(1)	0,639	WT basal: 0,73 ± 0,04	WT basal vs WT KCl	**p=0,000**
						WT KCl: 1,21 ± 0,07	WT basal vs DN basal	**p=0,016**
						DN basalt: 0,98 ± 0,03	WT basal vs DN KCl	**p=0,000**
						DN KCl: ± 1,31 ± 0,06	DN basal vs WT KCl	p=0,643
							DN basal vs DN KCl	**p=0,002**
							WT KCl vs DN KCl	p=0,319
Figure references	(1) Friedman Test					0,05		n = 36 ROIs , N = 11 independent experiment Figure 2
	**Statistical test (within groups comparison)**					0,05		
	Pairwise comparison							
	**Parameter**	**Statistical result**	**p-value**	**Statistical test**	**Effect size (r)**	**Descriptive and pairwise comparison**		
						**groups**	**comparisons**	**Corrected p-value**
J	Ratio PM/C	$X^2(3)$=63,503	p=0,000	(1)	0,638	WT basal: 0,68 ± 0,03	WT basal vs WT KCl	**p=0,000**
						WT KCl: 1,16 ± 0,04	WT basal vs 6A basal	**p=0,000**
						6A basalt: 0,93 ± 0,05	6A basal vs 6A KCl	p=1
						6A KCl: ± 0,99 ± 0,07	WT basal vs 6A KCl	**p=0,000**
							WT KCl vs6A KCl	**p=0,001**
							WT KCl vs 6A basal	p=0,001

Figure 3. Ca2+ binding site mutations cause constitutive phospholipid binding of C2A and C2AB fragments of Doc2b

Figure references	Statistical test (between groups comparison)					Accepted α-significance		Sample
	(2) Kruskal-Wallis Test					**0,05**		n =15 , N = 5 independent experiment
	**Parameter**	**Statistical result**	**p-value**	**Statistical test**	**Effect size (r)**	**Descriptive and pairwise comparison**		
						**groups**	**comparisons**	**Corrected p-value**
H	[Ca2+]free = 2,2 nM	H(2)=11,580	**p=0,003**	(2)	2,990			
	[Ca2+]free = 7 nM	H(2)=9,620	**p=0,008**	(2)	2,484			
	[Ca2+]free = 36 nM	H(2)=10,288	**p=0,006**	(2)	2,656			
	[Ca2+]free = 163 nM	H(2)=10,593	**p=0,005**	(2)	2,735			
	[Ca2+]free = 509 nM	H(2)=6,228	p=0,044	(2)	n.a.			
	[Ca2+]free = 655 nM	H(2)=14,332	**p=0,001**	(2)	3,700			
	[Ca2+]free = 834 nM	H(2)=11,863	**p=0,003**	(2)	3,063			
	[Ca2+]free = 1075 nM	H(2)=11,580	**p=0,003**	(2)	3,000			
	[Ca2+]free = 2637 nM	H(2)=11,580	**p=0,003**	(2)	3,000			
	[Ca2+]free = 10941 nM	H(2)=11,580	**p=0,003**	(2)	3,000			
Figure reference	**Statistical test (between groups comparison)**					**Accepted α-significance**		**Sample**
	(2) Kruskal-Wallis Test					**0,05**		n =12 , N = 4 independent
	**Parameter**	**Statistical result**	**p-value**	**Statistical test**	**Effect size (r)**	**Descriptive and pairwise comparison**		
						**groups**	**comparisons**	**Corrected p-value**
P	[Ca2+]free = 2,2 nM	H(2)=8,346	**p=0,015**	(2)	2,409			
	[Ca2+]free = 7 nM	H(2)=9,269	**p=0,010**	(2)	2,676			
	[Ca2+]free = 36 nM	H(2)=8,346	**p=0,015**	(2)	2,409			
	[Ca2+]free = 163 nM	H(2)=6,500	**p=0,039**	(2)	1,876			
	[Ca2+]free = 509 nM	H(2)=9,846	**p=0,007**	(2)	2,842			
	[Ca2+]free = 655 nM	H(2)=7,423	**p=0,024**	(2)	2,143			
	[Ca2+]free = 834 nM	H(2)=8,769	**p=0,012**	(2)	2,531			
	[Ca2+]free = 1075 nM	H(2)=7,538	**p=0,023**	(2)	2,176			
	[Ca2+]free = 2637 nM	H(2)=4,154	p=0,125	(2)	n.a.			
	[Ca2+]free = 10941 nM	H(2)=3,500	p=0,174	(2)	n.a.			

Figure 4. Doc2b Ca^{2+}-binding-site mutants enhance the frequency of spontaneous release in Doc2-deficient hippocampal neurons

Figure references	Statistical test (between groups comparison)	Accepted α-significance	Sample
	(1) Kruskal-Wallis Test	**0.01**	n = 197, N = 5 independent experiment
	**Statistical test (within groups comparison)**	0.05	
	Pairwise comparison		

Figure references	Parameter	Statistical result	p-value	Statistical test	Effect size (r)	groups	comparisons	Corrected p-value
B	mEPSC frequency	H(3)=64,111	**p=0,000**	(1)	4,568	eGFP*: 10,01 ± 1,424	eGFP vs WT	p=1
						WT*: 13,27 ± 1,568	eGFP vs DN	**p=0,000**
						DN*: 28,08 ± 3,226	eGFP vs 6A	**p=0,000**
						6A*: 35,23 ± 2,188	WT vs DN	**p=0,002**
							WT vs 6A	**p=0,000**
							DN vs 6A	p=0,095
C	mEPSC amplitude	H(3)=1,465	p=0,690	(1)	n.a.			
D	mEPSC charge	H(3)=11,767	**p=0,008**	(1)	0,838	eGFP*: 89,35 ± 8,384	eGFP vs WT	p=1
						WT*: 85,44 ± 8,168	eGFP vs DN	p=1
						DN*: 86,10 ± 9,856	eGFP vs 6A	p=0,591
						6A*: 123,65 ± 10,402	WT vs DN	p=1
							WT vs 6A	**p=0,016**
							DN vs 6A	**p=0,027**
E	mEPSC rise	H(3)=3,599	p=0,308	(1)	n.a.			
F	mEPSC decay	H(3)=6,849	p=0,077	(1)	n.a.			

Figure 5. Ca^{2+} binding site mutants Doc2bDN and Doc2b6A enhance short-term depression in Doc2-deficient neurons

Figure references	Statistical test (between groups comparison)	Accepted α-significance	Sample
	(1) Kruskal-Wallis Test	**0,025**	n = 139, N = 5 independent experiment
	**Statistical test (within groups comparison)**	0.05	
	Pairwise comparison		

Figure references	Parameter	Statistical result	p-value	Statistical test	Effect size (r)	groups	comparisons	Corrected p-value
B	1st EPSC amplitude	H(3)=6,481	p=0,090	(1)	n.a.			
C	1st EPSC charge	H(3)=6,279	p=0,099	(1)	n.a.			

Figure references	Statistical test (between groups comparison)	Accepted α-significance	Sample
	(1) Kruskal-Wallis Test	**0,025**	n = 102, N = 5 independent experiment
	**Statistical test (within groups comparison)**	0.05	
	Pairwise comparison		

Figure references	Parameter	Statistical result	p-value	Statistical test	Effect size (r)	groups	comparisons	Corrected p-value
D	1st EPSC quantal content	H(3)=3,476	p=0,324	(1)	n.a.			

Figure references	Statistical test (between groups comparison)	Accepted α-significance	Sample
	(1) One-way repeated measures ANOVA<br>(2) Kruskal-Wallis Test	**0,008**	n = 139, N = 5 independent experiment
	**Statistical test (within groups comparison)**	0.05	
	Pairwise comparison		

Figure references	Parameter	Statistical result	p-value	Statistical test	Effect size (r)	groups	comparisons	Corrected p-value
F	PP ratio 1	H(3)=19,818	**p=0,000**	(2)	1,681	eGFP*: 0,90 ± 0,063	eGFP vs WT	p=1
						WT*: 0,78 ± 0,039	eGFP vs DN	**p=0,001**
						DN*: 0,66 ± 0,030	eGFP vs 6A	**p=0,002**
						6A*: 0,68 ± 0,025	WT vs DN	p=0,057
							WT vs 6A	p=0,146
							DN vs 6A	p=1
F	PP ratio 2	H(3)=23,150	**p=0,000**	(2)	1,964	eGFP*: 0,92 ± 0,036	eGFP vs WT	p=1
						WT*: 0,84 ± 0,034	eGFP vs DN	**p=0,000**
						DN*: 0,74 ± 0,029	eGFP vs 6A	**p=0,002**
						6A*: 0,75 ± 0,028	WT vs DN	**p=0,014**
							WT vs 6A	p=0,083
							DN vs 6A	p=1
F	PP ratio 3	F(3,75)=8,465	**p=0,000**	(1)	0,859	eGFP*: 0,93 ± 0,038	eGFP vs WT	p=0,456
						WT*: 0,86 ± 0,035	eGFP vs DN	**p=0,013**
						DN*: 0,75 ± 0,026	eGFP vs 6A	**p=0,000**
						6A*: 0,74 ± 0,022	WT vs DN	p=0,475
							WT vs 6A	p=0,084
							DN vs 6A	p=1
F	PP ratio 4	H(3)=20,454	**p=0,000**	(2)	1,735	eGFP*: 0,89 ± 0,040	eGFP vs WT	p=1
						WT*: 0,84 ± 0,031	eGFP vs DN	**p=0,002**
						DN*: 0,75 ± 0,026	eGFP vs 6A	**p=0,003**
						6A*: 0,77 ± 0,017	WT vs DN	**p=0,023**
							WT vs 6A	**p=0,042**
							DN vs 6A	p=1
F	PP ratio 5	H(3)=22,178	**p=0,000**	(2)	1,881	eGFP*: 0,89 ± 0,026	eGFP vs WT	p=1
						WT*: 0,87 ± 0,026	eGFP vs DN	**p=0,010**
						DN*: 0,77 ± 0,022	eGFP vs 6A	**p=0,009**
						6A*: 0,78 ± 0,015	WT vs DN	**p=0,003**
							WT vs 6A	**p=0,002**
							DN vs 6A	p=1
F	PP ratio 6	H(3)=32,730	**p=0,000**	(2)	2,776	eGFP*: 0,89 ± 0,029	eGFP vs WT	p=0,488
						WT*: 0,91 ± 0,025	eGFP vs DN	p=0,055
						DN*: 0,80 ± 0,021	eGFP vs 6A	**p=0,015**
						6A*:0,80 ± 0,014	WT vs DN	**p=0,000**
							WT vs 6A	**p=0,000**
							DN vs 6A	p=1

Figure 6. Doc2b mutants affect synaptic strength rundown during repetitive stimulations in DKO neurons

Figure references	Statistical test (between groups comparison)					Accepted α-significance		Sample
	(1) Kruskal-Wallis Test					**0,0167**		n = 107, N = 5 independent experiment
	**Statistical test (within groups comparison)**					0.05		
	Pairwise comparison							
	**Parameter**	**Statistical result**	**p-value**	**Statistical test**	**Effect size (r)**	**Descriptive and pairwise comparison**		
						**groups**	**comparisons**	**Corrected p-value**
E	Cumulative total charge	H(3)=2,380	p=0,497	(1)	n.a.			
F	Cumulative normalized charge	H(3)=27,255	**p=0,000**	(1)	2,664	eGFP*: 59,94 ± 5,61	eGFP vs WT	p=0,606
						WT*: 70,91 ± 5,07	eGFP vs DN	p=0,338
						DN*: 44,72 ± 2,25	eGFP vs 6A	**p=0,015**
						6A*:39,30 ± 1,85	WT vs DN	**p=0,002**
							WT vs 6A	**p=0,000**
							DN vs 6A	p=1
G	Synchronous & Asynchronous charge proportion	H(3)=10,386	**p=0,016**	(1)	1,005	eGFP*: 0,83 ± 0,013 vs 0,17 ± 0,013	eGFP vs WT	**p=0,013**
						WT*: 0,76 ± 0,014 vs 0,24 ± 0,014	eGFP vs DN	p=0,391
						DN*: 0,79 ± 0,022 vs 0,21 ± 0,022	eGFP vs 6A	p=1
						6A*: 0,81 ± 0,016 vs 0,19 ± 0,016	WT vs DN	p=1
							WT vs 6A	p=0,263
							DN vs 6A	p=1
H	Recovery 5Hz	H(3)=10,714	**p=0,013**	(1)	1,036	eGFP*: 0,52 ± 0,059	eGFP vs WT	p=0,217
						WT*: 0,60 ± 0,049	eGFP vs DN	p=1
						DN*: 0,48 ± 0,031	eGFP vs 6A	p=1
						6A*: 0,42 ± 0,024	WT vs DN	p=0,221
							WT vs 6A	**p=0,013**
							DN vs 6A	p=1

Figure references	Statistical test (between groups comparison)					Accepted α-significance		Sample
	(1) Kruskal-Wallis Test					**0,0167**		n = 101, N = 5 independent experiment
	**Statistical test (within groups comparison)**					0.05		
	Pairwise comparison							
	**Parameter**	**Statistical result**	**p-value**	**Statistical test**	**Effect size (r)**	**Descriptive and pairwise comparison**		
						**groups**	**comparisons**	**Corrected p-value**
M	Cumulative total charge	H(3)=1,082	p=0,781	(1)	n.a.			
N	Cumulative normalized charge	H(3)=19,425	**p=0,000**	(1)	1,933	eGFP*: 43,34 ± 3,69	eGFP vs WT	p=1
						WT*: 45,85 ± 3,40	eGFP vs DN	p=0,114
						DN*: 33,88 ± 4,75	eGFP vs 6A	**p=0,024**
						6A*: 29,20 ± 3,09	WT vs DN	**p=0,010**
							WT vs 6A	**p=0,001**
							DN vs 6A	p=1
O	Synchronous & Asynchronous charge proportion	H(3)=7,705	p=0,053	(1)	n.a.			
P	Recovery 40Hz	H(3)=16,682	**p=0,001**	(1)	1,660	eGFP*: 0,81 ± 0,064	eGFP vs WT	p=0,181
						WT*: 0,99 ± 0,076	eGFP vs DN	p=1
						DN*: 0,076 ± 0,061	eGFP vs 6A	p=0,738
						6A*: 0,68 ± 0,030	WT vs DN	**p=0,025**
							WT vs 6A	**p=0,001**
							DN vs 6A	p=1
R	RRP size (pC)	H(3)=1,073	p=0,784	(1)	n.a.			
S	Release probability	H(3)=11,453	**p=0,010**	(1)	1,150	eGFP*: 0,349 ± 0,032	eGFP vs WT	p=1
						WT*: 0,430 ± 0,050	eGFP vs DN	p=0,348
						DN*: 0,472 ± 0,051	eGFP vs 6A	**p=0,010**
						6A*: 0,560± 0,051	WT vs DN	p=1
							WT vs 6A	p=0,067
							DN vs 6A	p=1

Figure 7. Overexpression of Doc2bWT, Doc2bDN, and Doc2b6A does not affect morphology or synapse number in wildtype neurons

Statistical test (between groups comparison)	Accepted α-significance	Sample
(1) One-way repeated measures ANOVA		n = 168, N = 3 independent experiment
(2) Kruskal-Wallis Test	**0,01**	
**Statistical test (within groups comparison)**	0.05	
Pairwise comparison		

Figure references	Parameter	Statistical result	p-value	Statistical test	Effect size (r)	groups	comparisons	Corrected p-value
B	Synapse number	H(3)=3,452	p=0,327	(2)	n.a.			
B	Dendritic length	F(3,117)=0,465	p=0,707	(1)	n.a.			
B	Synapses/µm	H(3)=5,247	p=0,155	(2)	n.a.			
B	Synapse area	H(3)=4,883	p=0,181	(2)	n.a.			
B	Soma area	H(3)=0,476	p=0,924	(2)	n.a.			

(The groups, comparisons and Corrected p-value columns are under the heading "Descriptive and pairwise comparison".)

Figure S2. Doc2b^{DN} and Doc2b^{6A} mutations enhance the frequency of spontaneous release in wildtype hippocampal neurons

Statistical test (between groups comparison)	Accepted α-significance	Sample
(1) Kruskal-Wallis Test	**0.01**	n = 183, N = 4 independent experiment
**Statistical test (within groups comparison)**	0.05	
Pairwise comparison		

Figure references	Parameter	Statistical result	p-value	Statistical test	Effect size (r)	groups	comparisons	Corrected p-value
B	mEPSC frequency	H(3)=73,308	**p=0,000**	(1)	5,863	eGFP*: 4,33 ± 0,544	eGFP vs WT	p=1
						WT*: 4,64 ± 0,628	eGFP vs DN	**p=0,000**
						DN*: 15,67 ± 1,830	eGFP vs 6A	**p=0,000**
						6A*: 16,51 ± 1,842	WT vs DN	**p=0,000**
							WT vs 6A	**p=0,000**
							DN vs 6A	p=1
C	mEPSC amplitude	H(3)=1,851	p=0,604	(1)	n.a.			
D	mEPSC area	H(3)=2,678	p=0,444	(1)	n.a.			
E	mEPSC rise	H(3)=1,937	p=0,586	(1)	n.a.			
F	mEPSC decay	H(3)=0,839	p=0,840	(1)	n.a.			

Statistical test (between groups comparison)	Accepted α-significance	Sample
(1) Kruskal-Wallis Test	**0.01**	n = 218, N = 6 independent experiment
**Statistical test (within groups comparison)**	0.05	
Pairwise comparison		

Figure references	Parameter	Statistical result	p-value	Statistical test	Effect size (r)	groups	comparisons	Corrected p-value
H	mEPSC frequency	H(3)=104,468	**p=0,000**	(1)	7,075	eGFP*: 10,62 ± 1,093	eGFP vs WT	p=1
						WT*: 9,71 ± 1,171	eGFP vs DN	**p=0,000**
						DN*: 31,90 ± 1,736	eGFP vs 6A	**p=0,000**
						6A*: 30,06 ± 2,300	WT vs DN	**p=0,000**
							WT vs 6A	**p=0,000**
							DN vs 6A	p=1
I	mEPSC amplitude	H(3)=2,407	p=0,492	(1)	n.a.			
J	mEPSC area	H(3)=4,373	p=0,224	(1)	n.a.			
K	mEPSC rise time	H(3)=9,060	p=0,029	(1)	0,614			
L	mEPSC decay Time	H(3)=18,609	**p=0,000**	(1)	1,260	eGFP*: 6,07 ± 0,127	eGFP vs WT	p=0,238
						WT*: 5,62 ± 0,137	eGFP vs DN	p=1
						DN*: 6,36 ± 0,185	eGFP vs 6A	p=0,327
						6A*: 6,64 ± 0,222	WT vs DN	**p=0,012**
							WT vs 6A	**p=0,000**
							DN vs 6A	p=1

(The groups, comparisons and Corrected p-value columns are under the heading "Descriptive and pairwise comparison".)

Figure S3. Doc2b mutants affect short term plasticity in WT autaptic neurons

Figure references	Statistical test (between groups comparison)					Accepted α-significance		Sample
	(1) One-way repeated measures ANOVA					**0,025**		n = 157, N = 6 independent experiment
	(2) Kruskal-Wallis Test							
	**Statistical test (within groups comparison)**					0.05		
	Pairwise comparison							
	**Parameter**	**Statistical result**	**p-value**	**Statistical test**	**Effect size (r)**	**Descriptive and pairwise comparison: groups**	**comparisons**	**Corrected p-value**
B	1st EPSC amplitude	F(3,111)= 1,427	p=0,239	(1)	n.a.			
C	1st EPSC charge	H(3)=11,041	**p=0,012**	(2)	0,881	eGFP*: 137,96 ± 12,691	eGFP vs WT	p=0,211
						WT*: 98,096 ± 10,515	eGFP vs DN	p=1
						DN*: 163,43 ± 17,967	eGFP vs 6A	p=1
						6A*: 174,28 ± 19,616	WT vs DN	**p=0,043**
							WT vs 6A	**p=0,013**
							DN vs 6A	p=1

Figure references	Statistical test (between groups comparison)					Accepted α-significance		Sample
	(1) One-way repeated measures ANOVA					**0,008**		n = 165, N = 6 independent experiment
	(2) Kruskal-Wallis Test							
	**Statistical test (within groups comparison)**					0.05		
	Pairwise comparison							
	**Parameter**	**Statistical result**	**p-value**	**Statistical test**	**Effect size (r)**	**Descriptive and pairwise comparison: groups**	**comparisons**	**Corrected p-value**
D	PP ratio 1	H(3)=21,561	**p=0,000**	(2)	1,680	eGFP*: 0,86 ± 0,037	eGFP vs WT	p=1
						WT*: 0,85 ± 0,031	eGFP vs DN	**p=0,013**
						DN*: 0,70 ± 0,029	eGFP vs 6A	**p=0,005**
						6A*: 0,70 ± 0,026	WT vs DN	**p=0,007**
							WT vs 6A	**p=0,003**
							DN vs 6A	p=1
D	PP ratio 2	F(3,108)=12,620	**p=0,000**	(1)	0,899	eGFP*: 1,01 ± 0,034	eGFP vs WT	p=1
						WT*: 0,96 ± 0,026	eGFP vs DN	**p=0,002**
						DN*: 0,81 ± 0,025	eGFP vs 6A	**p=0,000**
						6A*: 0,78 ± 0,024	WT vs DN	**p=0,006**
							WT vs 6A	**p=0,000**
							DN vs 6A	p=1
D	PP ratio 3	F(3,108)=15,793	**p=0,000**	(1)	0,917	eGFP*: 0,96 ± 0,020	eGFP vs WT	p=1
						WT*: 0,94 ± 0,022	eGFP vs DN	**p=0,000**
						DN*: 0,82 ± 0,017	eGFP vs 6A	**p=0,000**
						6A*: 0,78 ± 0,022	WT vs DN	**p=0,004**
							WT vs 6A	**p=0,000**
							DN vs 6A	p=1
D	PP ratio 4	H(3)=48,157	**p=0,000**	(2)	3,749	eGFP*: 0,94 ± 0,017	eGFP vs WT	p=1
						WT*: 0,91 ± 0,015	eGFP vs DN	**p=0,000**
						DN*: 0,82 ± 0,012	eGFP vs 6A	**p=0,000**
						6A*: 0,79 ± 0,019	WT vs DN	**p=0,000**
							WT vs 6A	**p=0,000**
							DN vs 6A	p=1
D	PP ratio 5	H(3)=40,339	**p=0,000**	(2)	3,140	eGFP*: 0,89 ± 0,017	eGFP vs WT	p=1
						WT*: 0,91 ± 0,014	eGFP vs DN	**p=0,003**
						DN*: 0,82 ± 0,011	eGFP vs 6A	**p=0,000**
						6A*: 0,80 ± 0,013	WT vs DN	**p=0,000**
							WT vs 6A	**p=0,000**
							DN vs 6A	p=1
D	PP ratio 6	H(3)=52,625	**p=0,000**	(2)	4,097	eGFP*: 0,89 ± 0,009	eGFP vs WT	p=0,158
						WT*: 0,94 ± 0,011	eGFP vs DN	**p=0,006**
						DN*: 0,84 ± 0,010	eGFP vs 6A	**p=0,000**
						6A*: 0,81 ± 0,014	WT vs DN	**p=0,000**
							WT vs 6A	**p=0,000**
							DN vs 6A	p=1

Figure references	Statistical test (between groups comparison)					Accepted α-significance		Sample
	(1) Kruskal-Wallis Test					**0,05**		n = 123, N = 6 independent experiment
	**Statistical test (within groups comparison)**					0.05		
	Pairwise comparison							
	**Parameter**	**Statistical result**	**p-value**	**Statistical test**	**Effect size (r)**	**Descriptive and pairwise comparison: groups**	**comparisons**	**Corrected p-value**
E	1st EPSC quantal content	H(3)=9,292	**p=0,026**	(1)	0,838	eGFP*: 0,89 ± 0,009	eGFP vs WT	p=0,933
						WT*: 0,94 ± 0,011	eGFP vs DN	p=0,175
						DN*: 0,84 ± 0,010	eGFP vs 6A	**p=0,009**
						6A*: 0,81 ± 0,014	WT vs DN	p=0,205
							WT vs 6A	**p=0,012**
							DN vs 6A	p=0,177

Figure S3. Doc2b mutants affect short term plasticity in WT autaptic neurons

Statistical test (between groups comparison)	Accepted α-significance	Sample
(1) Kruskal-Wallis Test	**0,0167**	n = 157, N = 6 independent experiment
**Statistical test (within groups comparison)**	0.05	
Pairwise comparison		

Figure references	Parameter	Statistical result	p-value	Statistical test	Effect size (r)	groups	comparisons	Corrected p-value
I	Cumulative total charge	H(3)=4,21	p=0,240	(1)	n.a.			
J	Cumulative normalized charge	H(3)=59,983	**p=0,000**	(1)	4,787	eGFP*: 61,51 ± 3,277	eGFP vs WT	p=0,292
						WT*: 72,13 ± 3,391	eGFP vs DN	**p=0,000**
						DN*: 44,23 ± 2,352	eGFP vs 6A	**p=0,000**
						6A*: 41,57 ± 2,121	WT vs DN	**p=0,000**
							WT vs 6A	**p=0,000**
							DN vs 6A	p=1
K	Synchronous & Asynchronous charge proportion	H(3)=1,977	p=0,577	(1)	n.a.			
L	Recovery 5Hz	H(3)=24,758	**p=0,000**	(1)	1,976	eGFP*: 0,52 ± 0,025	eGFP vs WT	**p=0,012**
						WT*: 0,67 ± 0,035	eGFP vs DN	p=1
						DN*: 0,50 ± 0,019	eGFP vs 6A	p=0,654
						6A*: 0,48 ± 0,018	WT vs DN	**p=0,000**
							WT vs 6A	**p=0,000**
							DN vs 6A	p=1

Statistical test (between groups comparison)	Accepted α-significance	Sample
(1) Kruskal-Wallis Test	**0,0167**	n = 144, N = 6 independent experiment
**Statistical test (within groups comparison)**	0.05	
Pairwise comparison		

Figure references	Parameter	Statistical result	p-value	Statistical test	Effect size (r)	groups	comparisons	Corrected p-value
P	Cumulative total charge	H(3)=4,868	p=0,182	(1)	n.a.			
Q	Cumulative normalized charge	H(3)=42,608	**p=0,000**	(1)	3,551	eGFP*: 39,83 ± 2,265	eGFP vs WT	p=1
						WT*: 45,07 ± 3,634	eGFP vs DN	**p=0,000**
						DN*: 24,86 ± 1,491	eGFP vs 6A	**p=0,000**
						6A*: 26,08 ± 1,821	WT vs DN	**p=0,000**
							WT vs 6A	**p=0,000**
							DN vs 6A	p=1
R	Synchronous & Asynchronous charge proportion	H(3)=8,082	**p=0,044**	(1)	0,674	eGFP*: 0,14 ± 0,008 vs 0,86 ± 0,008	eGFP vs WT	p=0,894
						WT*: 0,15 ± 0,007 vs 0,85 ± 0,007	eGFP vs DN	**p=0,027**
						DN*: 0,18 ± 0,012 vs 0,82 ± 0,012	eGFP vs 6A	p=0,893
						6A*: 0,17 ±0,013 vs 0,83 ± 0,013	WT vs DN	p=1
							WT vs 6A	p=1
							DN vs 6A	p=0,991
S	Recovery 40Hz	H(3)=52,978	**p=0,000**	(1)	4,415	eGFP*: 0,76 ± 0,035	eGFP vs WT	**p=0,000**
						WT*: 1,15 ± 0,063	eGFP vs DN	p=1
						DN*: 0,70 ± 0,029	eGFP vs 6A	**p=0,038**
						6A*: 0,62 ± 0,028	WT vs DN	**p=0,000**
							WT vs 6A	**p=0,000**
							DN vs 6A	p=0,816
T	RRP size (pC)	H(3)=2,489	p=0,477	(1)	n.a.			
U	Release probability	H(3)=23,666	p=0,000	(1)	1,980	eGFP*: 0, 319 ± 0,023	eGFP vs WT	p=1
						WT*: 0,280 ± 0,021	eGFP vs DN	p=0,054
						DN*: 0,421 ± 0,031	eGFP vs 6A	**p=0,005**
						6A*: 0,430 ± 0,025	WT vs DN	**p=0,004**
							WT vs 6A	**p=0,000**
							DN vs 6A	p=1

Figure S4. Overexpression of wildtype Doc2b does not affect calcimycin-induced EPSC charge

Statistical test (between groups comparison)	Accepted α-significance	Sample
(1) One-way repeated measures ANOVA	**0,05**	n = 54, N = 3 independent experiment
**Statistical test (within groups comparison)**	0.05	
Pairwise comparison		

Figure references	Parameter	Statistical result	p-value	Statistical test	Effect size (r)	groups	comparisons	Corrected p-value
C	1st EPSC amplitude	F(1,27)=0,035	p=0,854	(1)	n.a.			

Statistical test (between groups comparison)	Accepted α-significance	Sample
(2) Mann-Whitney test	**0,025**	n = 49, N = 3 independent experiment
**Statistical test (within groups comparison)**	0.05	
Pairwise comparison		

Figure references	Parameter	Statistical result	p-value	Statistical test	Effect size (r)	groups	comparisons	Corrected p-value
D	Calcimycin peak amplitude	U=281	p=0,718	(2)	n.a.			
E	Calcimycin total charge(nC)	U=269	p=0,548	(2)	n.a.			
